# Dual pH-Responsive Calcium Phosphate Nanoparticles Conjugated with Folate by CuAAC Click Chemistry for Targeted Gemcitabine Delivery to Cancer Cells

**DOI:** 10.1021/acsabm.5c01683

**Published:** 2025-12-16

**Authors:** Thales R. Machado, Aileen Winter, Kateryna Loza, Kathrin Kostka, Valtencir Zucolotto, Matthias Epple

**Affiliations:** † Inorganic Chemistry, Centre for Nanointegration Duisburg-Essen (CENIDE), 119884 University of Duisburg-Essen, Essen 45117, Germany; ‡ GNANO − Nanomedicine and Nanotoxicology Group, São Carlos Institute of Physics, University of São Paulo, São Carlos, SP 13566-590, Brazil

**Keywords:** nanomedicine, nanotechnology, calcium phosphate, gemcitabine, folate, cancer treatment, targeted drug delivery

## Abstract

Calcium phosphate nanoparticles (CaP NPs) are biocompatible carriers widely studied for drug delivery due to their pH-responsive degradation and controlled release properties. In this study, CaP NPs stabilized with carboxymethyl cellulose (CMC) and coated with a silica layer were designed for gemcitabine (GEM) loading and folate (FA) conjugation, targeting cancer cells overexpressing folate receptor alpha (FRα). GEM was covalently coupled to CMC via an amide bond before CaP precipitation, creating a prodrug system. The NPs exhibited dual pH-responsive release, in which CaP dissolution combined with polymer-drug cleavage through acid-catalyzed hydrolysis of CMC-GEM within endolysosomes ensured intracellular bioavailability of free GEM molecules. FA conjugation by strong covalent bonds via copper-catalyzed azide–alkyne cycloaddition (CuAAC) click reaction enhanced the uptake of CaP NPs in FRα-positive breast cancer cells (MCF-7), whereas both FA-conjugated and nonconjugated NPs exhibited similar uptake in normal human mesenchymal stem cells (hMSCs). GEM-loaded CaP NPs showed high cytotoxicity in FRα-overexpressing cancer cell lines (MCF-7, MDA-MB-231, HeLa), while FA conjugation significantly reduced toxicity in hMSCs without compromising anticancer activity. These findings demonstrate the potential of FA-conjugated and GEM-loaded CaP NPs as a nanoplatform for targeted cancer therapy with reduced toxicity in healthy cells.

## Graphical abstract

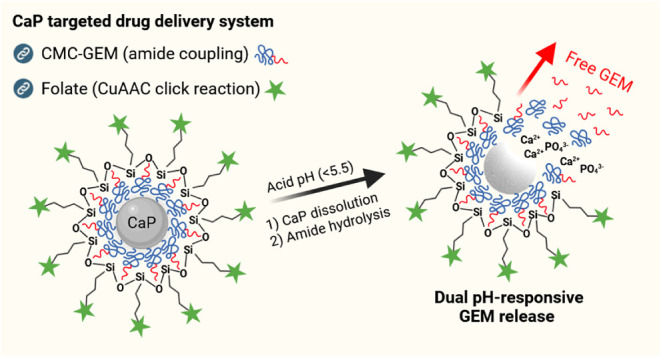

## 1 Introduction

Calcium phosphate nanoparticles (CaP NPs) are biocompatible and biodegradable nanomaterials that closely mimic the mineral phase of bone, making them ideal for a wide range of applications in tissue engineering and bone repair.^1^ Furthermore, their tunable size, surface charge, and composition allow precise control over their physicochemical properties, enabling the efficient loading of diverse molecules for targeted cancer therapy.^2–5^ A key feature of CaP NPs is their pH-responsive dissolution, which ensures stability in the bloodstream and promotes drug release in acidic tumor microenvironments or intracellular compartments like endolysosomes.^6^ Moreover, the dissolution elevates local Ca^2+^ and PO_4_^3–^ concentrations, increasing osmotic pressure that facilitates vesicle rupture and cargo release into the cytosol, thereby improving its bioavailability.^7^ The released ions can also disrupt Ca^2+^ homeostasis and trigger apoptosis, a mechanism particularly relevant in cancer cells, while healthy cells are more capable of restoring ionic balance, consequently reducing long-term toxicity compared to nondegradable NPs.^8^

CaP nanocarriers can be prepared by simple aqueous precipitation at room temperature, offering an accessible, cost-effective, and straightforward methodology that enables the incorporation of payloads during synthesis.^9,10^ However, their ionic nature does not permit a direct covalent functionalization, a crucial requirement to minimize premature cargo release. Besides that, CaP NPs coated with polymers such as carboxymethylcellulose (CMC) or polyethylenimine (PEI), further covered with a silica (SiO_2_) layer, exhibit promising surface chemistry that facilitates strong interactions with bioactive molecules before their pH-triggered release in target cells.^11,12^ Such CaP nanocarriers were used *inter alia* for efficient delivery of photosensitizers,^13,14^ siRNA and plasmid DNA,^15^ antibodies,^16^ or proteins.^17^ Additionally, the SiO_2_ layer enables a further conjugation step through bioorthogonal click chemistry, e.g., copper-catalyzed azide–alkyne cycloaddition (CuAAC). This reaction allows a robust covalent attachment of fluorescent dyes,^18^ bioactive ligands or ultrasmall metallic NPs.^19^

Here, we want to demonstrate the application of CaP-CMC/SiO_2_ nanocarriers for the delivery of antineoplastic molecules. For this purpose, we selected gemcitabine (GEM), a nucleoside analogue widely used as a first-line treatment for pancreatic, lung, breast, and bladder cancer, which acts by incorporating into DNA during replication, causing chain termination and apoptosis.^20^ Despite its clinical relevance, GEM undergoes rapid deamination by cytidine deaminase, has a short plasma half-life, and induces dose-limiting toxicity. To overcome these issues, some studies have explored the loading of GEM and its analogues on CaP NPs.^21–25^ The main challenge is that the neutral character of GEM at the pH used for CaP precipitation hinders electrostatic interactions, reducing both its adsorption and stability on the NPs. Our solution is to conjugate GEM molecules covalently to the CMC polymer via amide bonds formed at their 4-(N)-amino functional groups prior to the CaP synthesis. This strategy ensures efficient loading within CaP-CMC/SiO_2_ NPs and provides protection against enzymatic deamination. Furthermore, it maintains GEM as the inactive CMC-GEM prodrug until its release inside cells by acid-catalyzed hydrolysis of the amide bonds.^26,27^

On the other hand, folate (FA), also known as vitamin B9, is an essential nutrient involved in DNA synthesis, repair, and methylation, playing a crucial role in cell division and overall metabolic function.^28^ In nanomedicine, FA has been widely explored as a ligand on nanocarriers for targeted drug delivery in the treatment of melanoma,^29^ glioblastoma,^30^ breast cancer,^31^ ovarian cancer,^32^ and other malignancies, whose cells overexpress folate receptor α (FRα). Moreover, several examples of FA-functionalized CaP NPs designed for the targeted delivery of other drugs (e.g., doxorubicin, epirubicin) rather than GEM have been reported.^33–35^ Since FA is exposed on the surface of the NPs and its premature detachment can impair targeting efficiency, strong surface anchoring is required. Our strategy involves the covalent conjugation of terminal alkyne-functionalized FA to azide-modified SiO_2_ surfaces of GEM-loaded CaP NPs via CuAAC. This reaction yields highly stable 1,3-disubstituted triazole linkages,^18^ ensuring site-specific, robust, and long-lasting anchoring of FA on the NP surface.

## 2 Results and Discussion

Figure 1 shows a schematic illustration of the synthetic route used to prepare CaP NPs conjugated with GEM and FA both via covalent bonding. During the chemical precipitation of CaP NPs, CMC is commonly employed as a stabilizer.^11^ Prior to CaP formation, GEM was conjugated to the CMC polymer through EDC/NHS coupling, establishing an amide bond between the activated carboxy groups of CMC and the primary amine of GEM. Subsequently, CMC serves a dual function, stabilizing the CaP NPs while facilitating efficient drug loading during step 1 of the synthesis procedure. In step 2, a silica layer is deposited, further enhancing the stability of the NPs and yielding the sample CaP-CMC-GEM/SiO_2_ (abbreviated as CaP-GEM), while the sample CaP-CMC/SiO_2_ (abbreviated as CaP) is obtained when pure CMC is used. In step 3, azide groups are introduced onto the silica surface, enabling the CuAAC click reaction to conjugate FA molecules, resulting in the sample CaP-CMC-GEM/SiO_2_–FA (abbreviated as CaP-GEM-FA) and the sample CaP-CMC/SiO_2_–FA (abbreviated as CaP-FA) when pure CMC is used.

**Figure 1:**
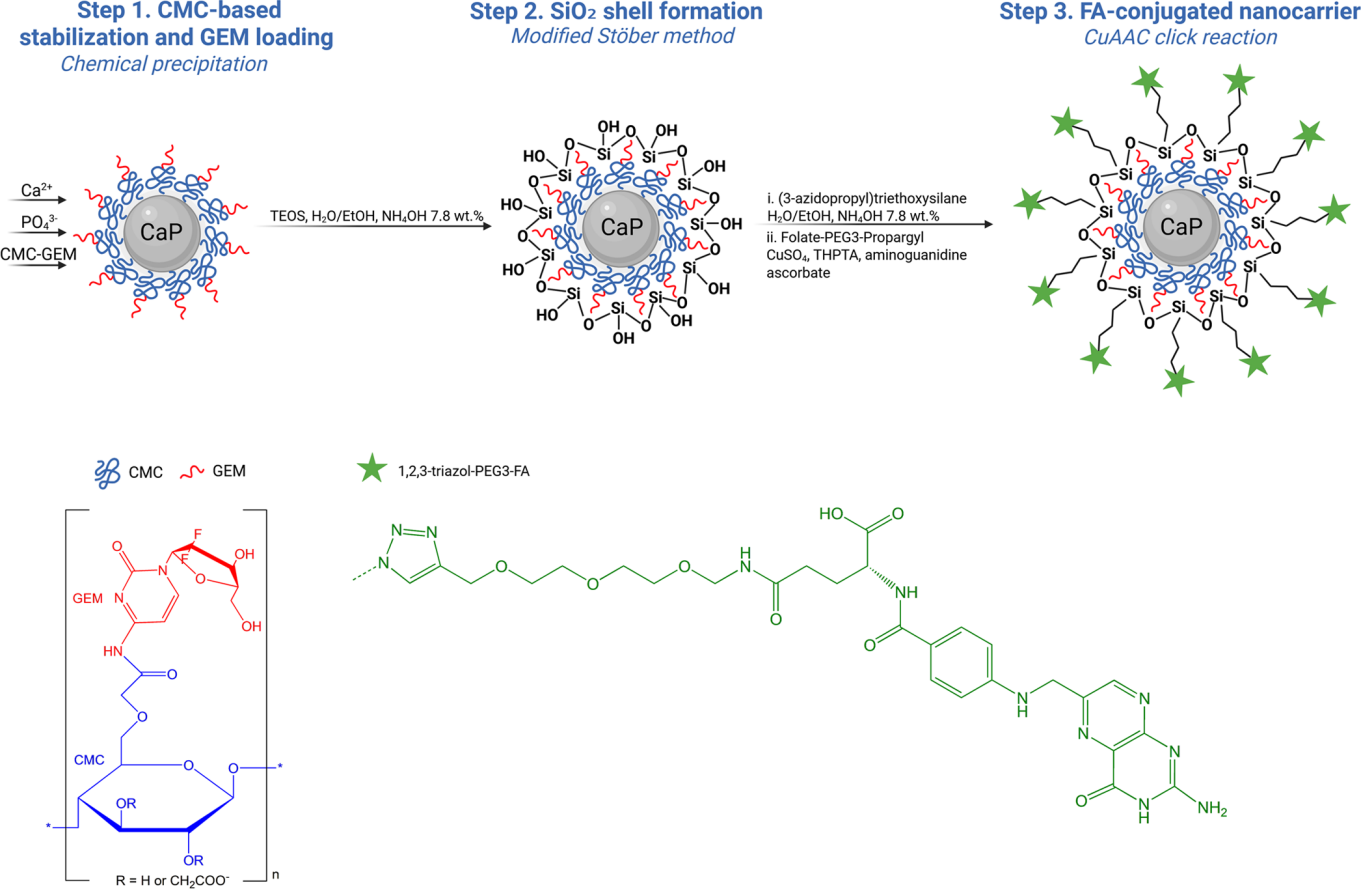
Schematic representation of the synthesis employed in this study to obtain FA- and GEM-conjugated CaP NPs stabilized by CMC polymer and a SiO_2_ layer.

### 2.1 CMC-GEM Characterization

The synthesis of the CMC-GEM conjugate and its characterization are detailed in Figure S1a–d of the (Supporting Information SI). FTIR analysis confirms the formation of new amide bonds between CMC and GEM (Figure S1b), while the protons from the pyrimidine ring of GEM are observed in the ^1^H NMR spectrum of CMC-GEM (Figure S1c). Additionally, fluorine atoms from the GEM molecule can be identified in ^19^F NMR (Figure S1d), confirming the successful conjugation. The chemical composition of CMC-GEM is presented in Table 1, while the calculation details and the UV–vis calibration curve used for its determination (Figure S2a) are provided in SI file. A substantial conjugation was achieved, with a GEM loading of 27.4 ± 0.5 μg per mg of CMC (2.74 wt %). Elemental analysis confirmed the presence of nitrogen atoms, further reinforcing the incorporation of the GEM molecule. Figure 2a presents the UV–vis spectra of CMC, GEM, and CMC-GEM in the range 200–400 nm. As expected, pure CMC gave no significant absorption, while GEM displays two characteristic absorption bands at 233 and 268 nm, corresponding to the π-π* electronic transition and the forbidden n-π* transition of the C = N group in the substituted pyrimidine ring.^36^ Following conjugation, the CMC-GEM spectrum showed two main absorption bands at 247 and 299 nm, indicating a significant influence of the CMC chains on the electronic density of the GEM chromophore groups, corroborating with NMR data.

**Table 1:** Summary of Conjugation Parameters and Chemical Composition of CMC-GEM Polymer

Parameter	Value
*w*(CMC)/mg mL^–1^	2.00
*w*(GEM) by UV–vis/μg mL^–1^	54.9 ± 0.9
*w*(GEM) per mg of CMC/μg mg^–1^	27.4 ± 0.5
CMC/GEM molar ratio	0.107 ± 0.002
Degree of substitution/DS^a^	0.03
C by elemental analysis/wt %	40.9 ± 0.1
H by elemental analysis/wt %	6.3 ± 0.2
O by elemental analysis/wt %	46.4 ± 0.9
N by elemental analysis/wt %	1.7 ± 0.1

a Extent of substitution of carboxyl groups on CMC by GEM molecules.

**Figure 2:**
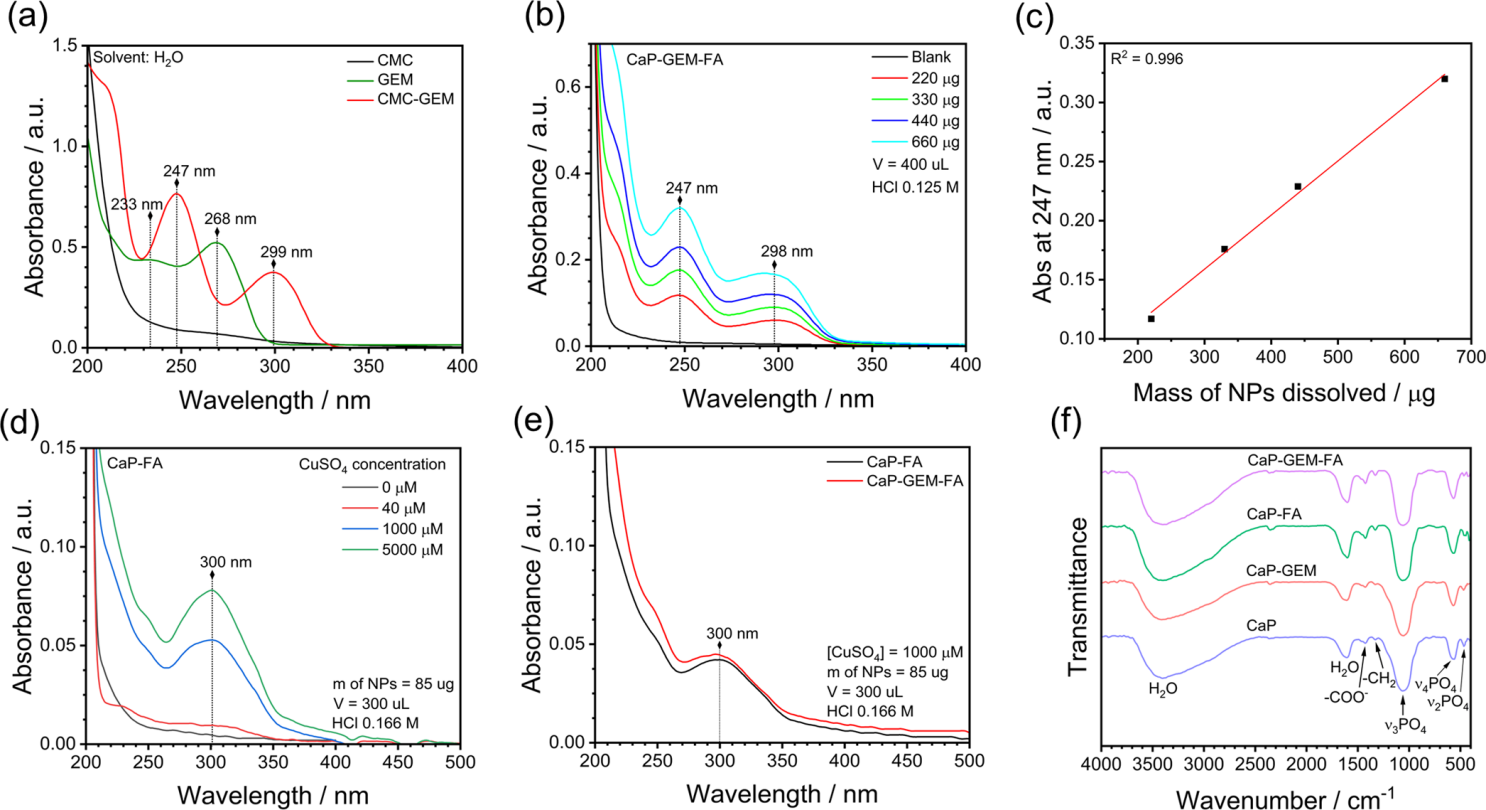
(a) UV–vis spectra of CMC (0.5 μg mL^–1^), GEM (16.4 μg mL^–1^), and CMC-GEM (0.5 μg mL^–1^ of CMC corresponding to 13.7 μg mL^–1^ of conjugated GEM). (b) UV–vis spectra of CaP-GEM-FA NPs dissolved in 0.125 M HCl before FA conjugation, using different sample masses (relative to the CaP core mass). (c) Linear correlation between the dissolved mass and the primary absorbance of CMC-GEM at 247 nm. The blank spectrum corresponds to CaP NPs synthesized in the presence of 1 mg mL^–1^ of free GEM. (d) UV–vis spectra of CaP NPs dissolved after the CuAAC click reaction with the FA precursor, performed with varying amounts of copper catalyst. (e) UV–vis spectra of CaP-FA and CaP-GEM-FA NPs synthesized via CuAAC click reaction using 1000 μM of copper catalyst. (f) FTIR spectra recorded after each synthesis step.

### 2.2 GEM- and FA-Conjugated CaP NPs Characterization

The GEM loading on CaP NPs was estimated using a direct UV–vis method, with the corresponding spectra shown in Figure 2b. The detailed calculations can be found in the SI file, along with the calibration curve used (Figure S2b). Upon dissolution of the NPs in an acidic solution, the two characteristic absorption bands of GEM conjugated to CMC were observed at 247 and 299 nm, corresponding to a loading of 1750 ± 250 GEM molecules per NP, or 3.3 ± 0.5 μg of GEM per mg of NPs (0.33 wt %), relative to the CaP core mass. Considering the degree of GEM conjugation to the CMC polymer, the estimated CMC loading on CaP NPs was 190 ± 25 chains of 90 kDa CMC per NP or 120 ± 20 μg of CMC per mg of NPs (12.0 wt %). As shown in Figure 2b,c, after removal of the supernatant containing unbound CMC-GEM, the dissolution of 220 μg, 330 μg, 440 μg, and 660 μg of NPs (based on the CaP core mass) resulted in a linear increase in absorbance at 247 nm, which confirms the presence of CMC-GEM on the surface of the CaP core. Additionally, a control experiment was conducted to assess simple adsorption, where a high excess of free GEM (1 mg mL^–1^) was added during chemical precipitation. The absence of significant UV–vis absorption upon dissolution of these NPs (Blank in Figure 2b) suggests negligible adsorption of free GEM onto the NPs.

After preparing the CaP and CaP-GEM NPs, the FA molecules were conjugated onto the nanoparticle surface via CuAAC click reaction. To optimize the conjugation conditions, different concentrations of the copper catalyst were tested, considering typical values for this reaction.^18,37^ Figure 2d shows the absorption spectra of CaP-FA NPs after the reaction with FA-PEG3-propargyl, following the dissolution of 85 μg of NPs in an acidic solution. In the absence of a copper catalyst (0 μM), the characteristic FA absorption band at 300 nm was not observed, eliminating possible nonspecific adsorption. At 40 μM, conjugation was minimal, while increasing the catalyst concentration to 1000 μM or 5000 μM resulted in a clear FA absorption band. This confirms the successful conjugation via the formation of 1,2,3-triazole linkages between the azide-functionalized NPs and the alkyne group of the FA precursor. To prevent potential NP aggregation caused by excess FA on the surface, we selected the NPs synthesized at 1000 μM for further studies. These CaP-FA NPs contained approximately 1200 ± 140 FA molecules per NP, corresponding to 3.5 ± 0.5 μg of FA per mg of NPs (0.35 wt %). Please refer to the SI file for detailed calculations, as well as the UV–vis absorption spectra and corresponding calibration curve (Figure S3a,b). Figure 2e compares the absorption spectra of CaP-FA and CaP-GEM-FA NPs, both synthesized with 1000 μM of the copper catalyst and dissolved in acid (85 μg, based on the CaP core mass). No significant differences were observed between the two samples, with GEM-containing NPs displaying an FA content of 1360 ± 160 molecules per NP or 4.0 ± 0.5 μg of FA per mg of NPs (0.40 wt %), a value similar to that of NPs without GEM. Table 2 provides a summary of all quantification results.

**Table 2:** Overview of the Quantification Results for All Prepared Samples

Parameter	CaP	CaP-GEM	CaP-FA	CaP-GEM-FA
CMC-GEM loading				
*w*(Ca^2+^) by AAS/μg mL^–1^	90	85	86	87
*w*(NPs)/μg mL^–1^	226	213	216	218
*N*(NPs)/NP mL^–1^	9.6 × 10^11^	9.1 × 10^11^	9.2 × 10^11^	9.3 × 10^11^
*w*(CMC) loaded by UV–vis/μg mL^–1^	-	30 ± 4	-	26 ± 4
*w*(CMC) per mg of NPs/μg mg^–1^	-	140 ± 20	-	120 ± 20
*N*(CMC) per NP/chain NP^–1^	-	220 ± 30	-	190 ± 25
*w*(GEM) loaded by UV–vis/μg mL^–1^	-	0.83 ± 0.12	-	0.72 ± 0.10
*w*(GEM) per mg of NPs/μg mg^–1^	-	4.0 ± 0.5	-	3.3 ± 0.5
*N*(GEM) per NP/molecule NP^–1^	-	2080 ± 300	-	1750 ± 250
GEM loading efficiency/%	-	8.0 ± 1.0	-	6.9 ± 1.0
FA conjugation				
*w*(Ca^2+^) by AAS/μg mL^–1^	-	-	40	40
*w*(NPs)/μg mL^–1^	-	-	100	100
*N*(NPs)/NP mL^–1^	-	-	4.3 × 10^11^	4.3 × 10^11^
*w*(FA) loaded by UV–vis/μg mL^–1^	-	-	0.35 ± 0.05	0.40 ± 0.05
*w*(FA) per mg of NPs/μg mg^–1^	-	-	3.5 ± 0.5	4.0 ± 0.5
*N*(FA) per NP/molecule NP^–1^	-	-	1200 ± 140	1360 ± 160

Figure 2f shows the FTIR spectra of CaP, CaP-GEM, CaP-FA, and CaP-GEM-FA NPs. In all cases, the spectra exhibit characteristic bands of CaP NPs, specifically the vibrational modes associated with distorted PO_4_^3–^ tetrahedra at 465 cm^–1^ (ν_2_PO_4_), 572 cm^–1^ (ν_4_PO_4_), and 1057 cm^–1^ (ν_3_PO_4_).^38^ This confirms that the structural integrity of the CaP cores was preserved throughout conjugation steps. Additionally, the absorption bands from CMC layer are observed at 1425 cm^–1^, and 1330 cm^–1^, corresponding to the symmetric stretching vibration of the carboxylate (−COO^–^) group and the C–H bending vibration of the methylene (−CH_2_) group, with contributions from the C–O stretching vibration of – COO^–^ group.^39^ Bands associated with GEM and FA are not detected by FTIR, probably due to the overlap of their fingerprint regions with the CaP bands.

The DLS analysis provides crucial insights into the hydrodynamic size, polydispersity (PDI), and surface charge of the CaP, CaP-GEM, CaP-FA, and CaP-GEM-FA NPs. As shown in Figure 3a, the hydrodynamic diameters by means of Z-average (Z-Avg) ranged from 168 to 196 nm. The low PDI values (Figure 3b) between 0.12 and 0.14 for all samples indicate a monodisperse and homogeneous distribution, confirming that the synthesis and functionalization processes were effective in maintaining well-controlled sizes in solution. As shown in Figure 3c, the zeta potentials of CaP and CaP-GEM were −24 mV and −25 mV, respectively, showing that GEM incorporation had a negligible effect on the surface charge. After FA functionalization, the zeta potential increased to −18 and −19 mV, reflecting a reduction in surface negativity due to the presence of FA functional groups partially neutralizing the negative charges. Despite this reduction, the values remain within a range indicative of good colloidal stability, as further supported by the size distributions shown in Figure S4.

**Figure 3:**
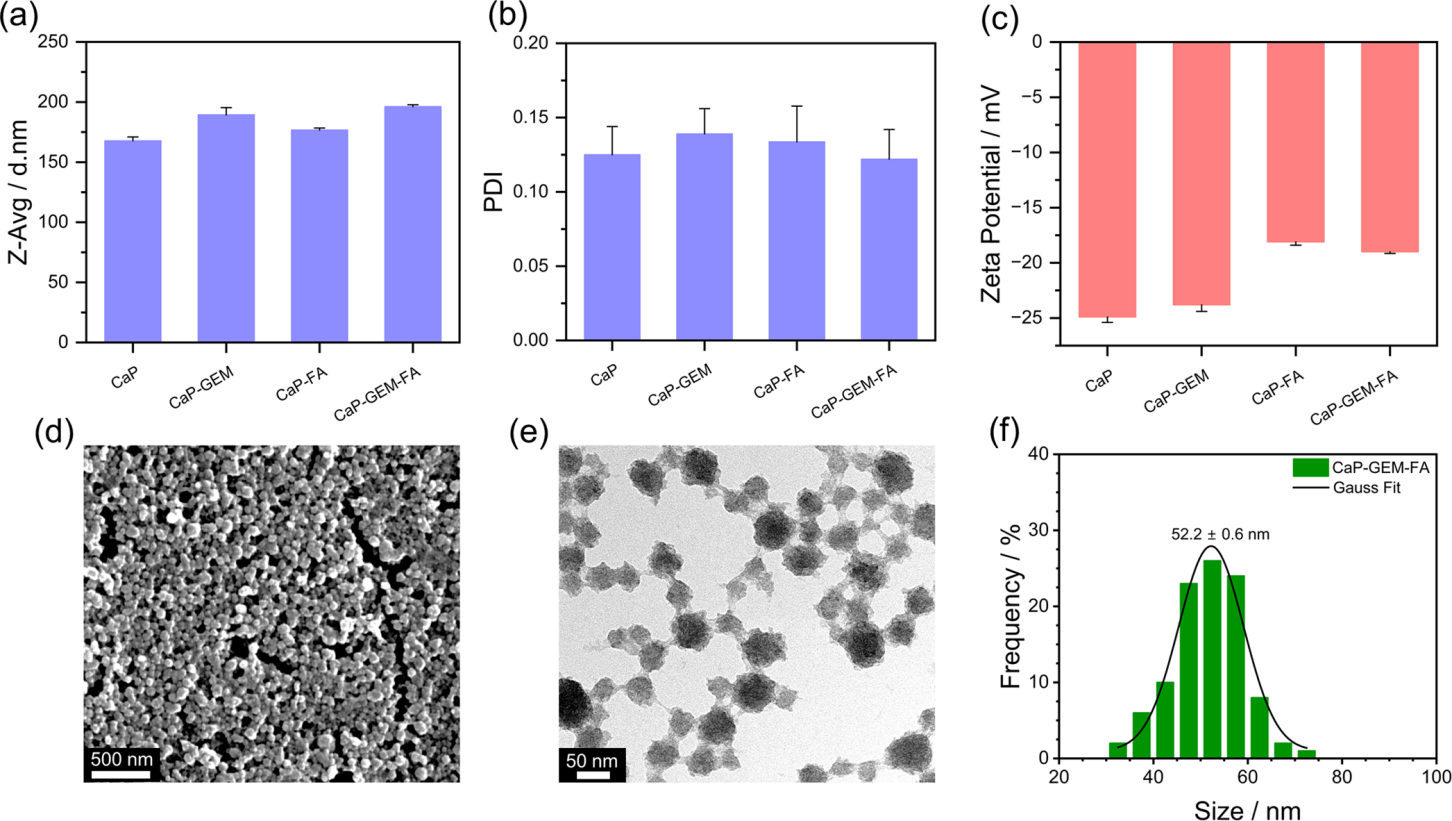
(a) Hydrodynamic diameter (Z-Avg) of all prepared CaP NPs, with the corresponding polydispersity index (PDI) and zeta potential values shown in (b) and (c). Electron microscopy characterization of CaP-GEM-FA NPs: (d) SEM image, (e) TEM image, and (f) CaP core size distribution.

The SEM and TEM analyses of the CaP-GEM-FA sample shown in Figure 3d–f revealed the presence of spherical NPs with a CaP core diameter of 52.2 nm, which is consistent with typical CaP-based nanostructures. Additionally, EDS analysis identified the main elements of the CaP phase, calcium and phosphorus, as well as silicon from the SiO_2_ shell (Figure S5). The calculated Ca/P ratio was 1.63, which aligns with the typical values found in CaP phases.^40^ The difference between the CaP core diameter and the hydrodynamic diameter suggests a moderate agglomeration of the NPs in aqueous dispersion. The TEM images presented in Figure S6a–d indicate that all prepared samples exhibited comparable morphology and CaP core sizes, while the slight variations observed in Z-Avg are attributed to minor differences in the degree of dispersion in solution.

### 2.3 Stability and Protein Corona Formation

The formation of a protein corona on NPs may alter their colloidal stability, circulation kinetics, and cellular internalization, thereby impacting their biological performance in delivery systems.^41^ In this study, the stability and protein corona formation on CaP-GEM-FA NPs were evaluated by DLS over a 72 h incubation at 37 °C in DMEM supplemented with 10% FBS, and compared with NPs dispersed in H_2_O. The corresponding results are presented in Figure 4a–c. An initial decrease in Z-Avg from 190 ± 5 nm in H_2_O to 130 ± 6 nm in DMEM + 10% FBS was observed, after which the size remains relatively constant throughout the experiment. The PDI values were relatively similar in both media, whereas slightly less negative zeta potential is noted compared to the CaP-GEM-FA NPs suspended in water. Figure 4d presents the FTIR spectra of the samples at the end of the experiment, confirming the structural integrity of the amorphous CaP phase over the probed period, as well as protein adsorption from FBS, evidenced by characteristic bands in the 1700–1400 cm^–1^ region.^42^ These findings demonstrate that the protein corona formation does not impair the colloidal stability of the NPs in the presence of FBS-derived proteins. Indeed, the adsorbed proteins may contribute to additional stabilization, possibly by interfering with interactions between adjacent CaP particles.

**Figure 4:**
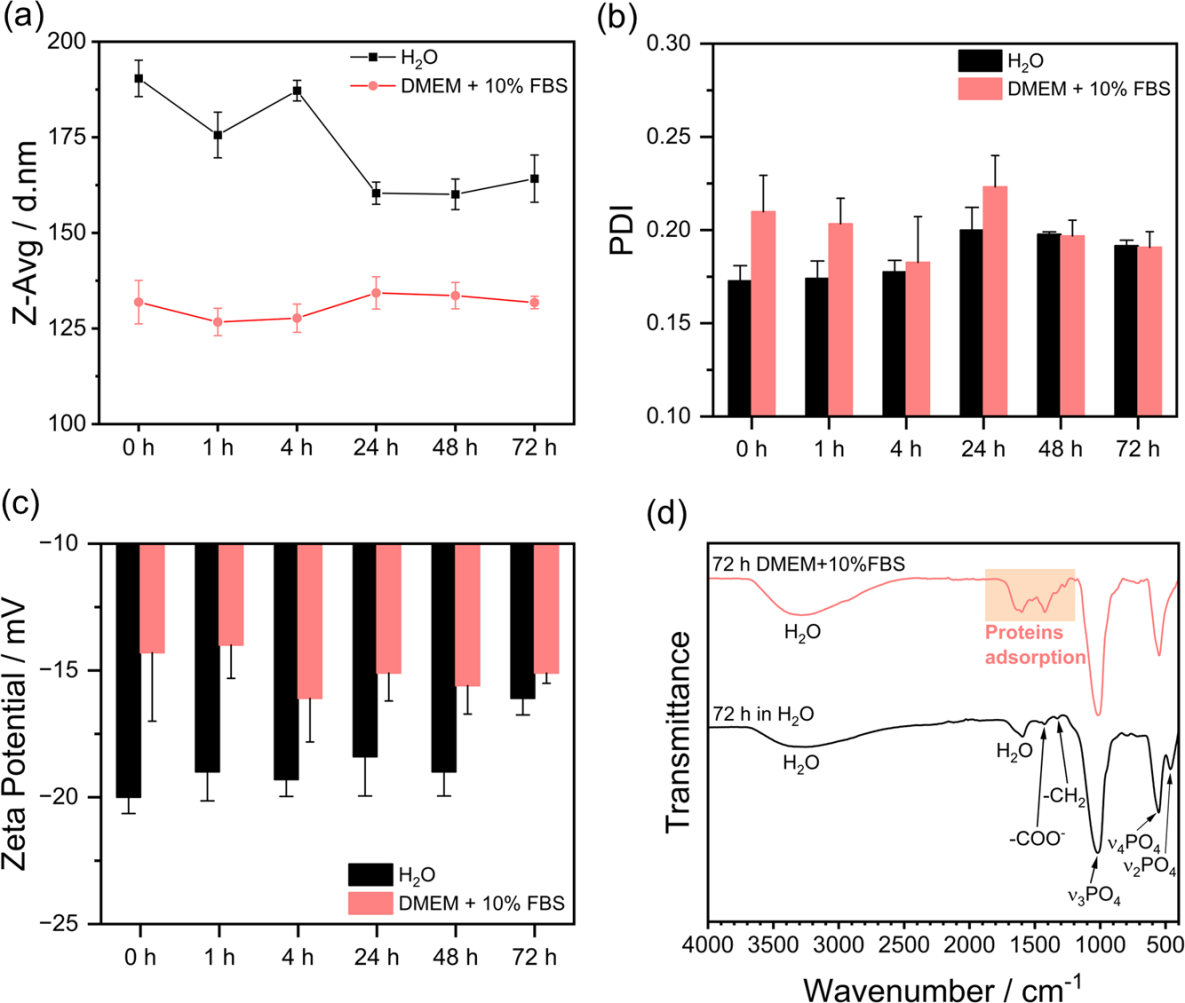
Stability and protein corona formation on CaP NPs over time at 37 °C. (a) Hydrodynamic diameter (Z-Avg), (b) PDI, and (c) zeta potential of CaP in water or DMEM with 10% FBS. (d) FTIR spectra after 72 h of incubation.

### 2.4 Kinetics of GEM and Ca^2+^ Release

The controlled release of loaded drugs is a crucial factor for the effectiveness of NPs in cancer treatment. In this study, HEPES buffer at pH 7.4 was used to mimic the physiological conditions of blood circulation, while acetate buffer at pH 4.5 simulated the acidic environment of endolysosomes, where drug release is often triggered.^43^ The release of GEM from CaP NPs demonstrated a typical sustained and pH-responsive profile, with gradual drug release at lower pH. As shown in Figure 5a, the cumulative release of GEM from CaP NPs at pH 7.4 was only 20% over 24 h, indicating that the NPs effectively minimized a burst release. After 72 h, the cumulative release was 26%, and by 192 h, the total release reached only 30%, ensuring that the drug remains significantly stable on the NPs under bloodstream conditions. This stability prevents premature elimination of the drug before reaching the target and reduces the probability of drug degradation in circulation. In contrast, when the pH was reduced to simulate an acidic environment, the GEM release from the NPs dramatically increased, reaching 50% at 24 h, 90% at 72 h, and ultimately 100% by the end of the experiment at 192 h. This pH-triggered release profile demonstrates the ability of the CaP NPs to control the release of GEM in response to the acidic conditions of tumor cells or endolysosomal compartments and to sustain a controlled release overtime.

**Figure 5:**
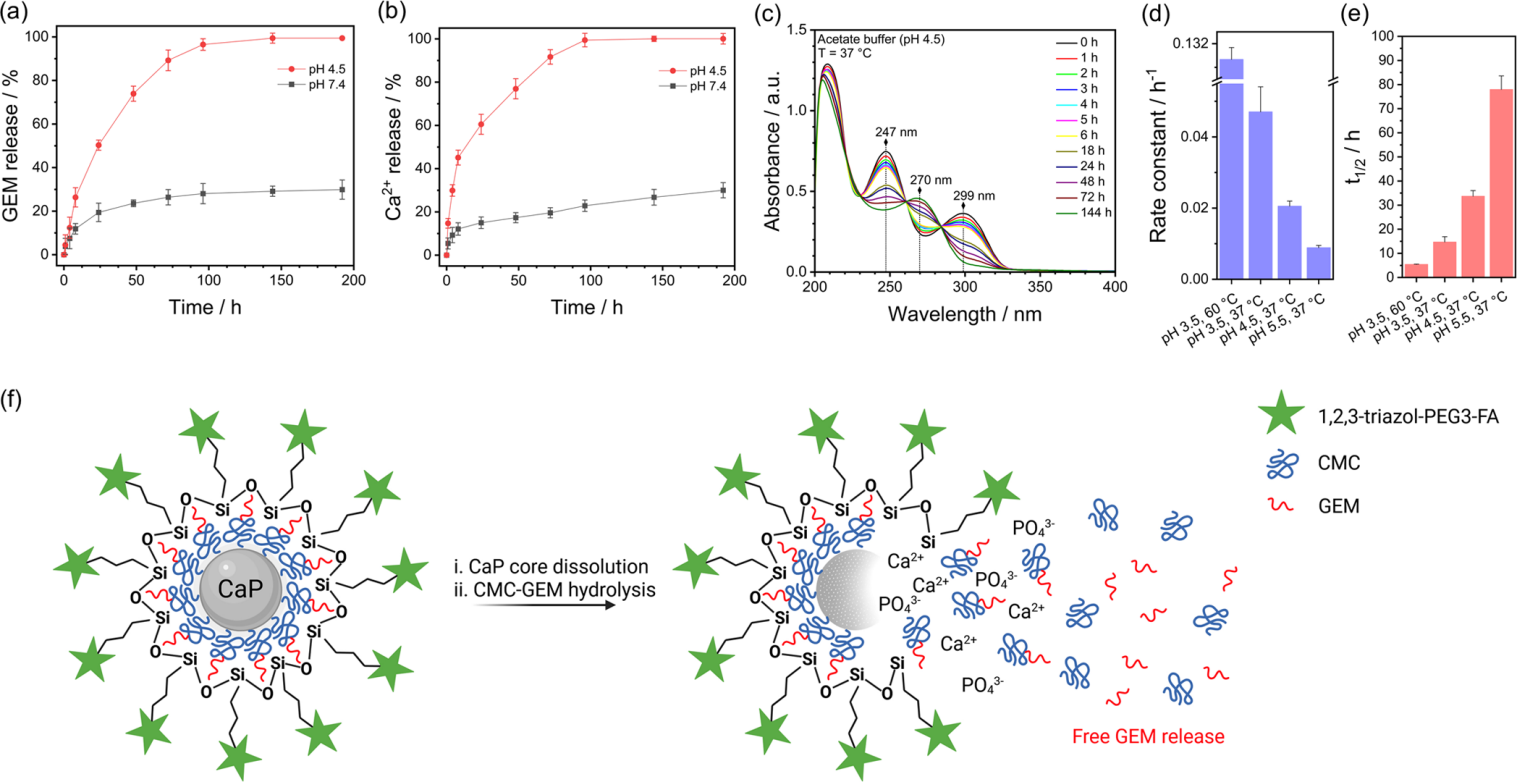
Kinetic results of the pH-responsive release of GEM and Ca^2+^, as well as the acid-catalyzed hydrolysis of the CMC-GEM conjugate polymer. (a) GEM and (b) Ca^2+^ release profiles at pH 7.4 (HEPES buffer) and pH 4.5 (acetate buffer) at 37 °C. (c) UV–vis spectra of CMC-GEM over time at 37 °C and pH 4.5. (d) Rate constants and (e) half-life times of the hydrolysis reaction, assuming a pseudo-first-order reaction under different temperature and pH conditions in acetate buffer. (f) Proposed scheme illustrating the pH-responsive behavior of CaP NPs, highlighting the simultaneous dissolution of the CaP core under acidic conditions and the release of free GEM via acid-catalyzed hydrolysis of CMC-GEM.

To further validate the pH-responsive drug release features of our nanocarrier, the same aliquots used in the drug release experiments were analyzed by AAS to quantify the concentration of Ca^2+^ released, serving as an indicator of CaP core degradation overtime. As shown in Figure 5b, the Ca^2+^ release profiles closely aligned with those of GEM, confirming a degradation-driven drug release. Notably, a significant increase in Ca^2+^ release was observed under acidic conditions, providing strong evidence that the CaP NPs undergo substantial degradation at lower pH, which justifies the enhanced drug release in these conditions.

### 2.5 Kinetics of CMC-GEM Acid Hydrolysis

Once the CaP core dissolves in an acidic environment, the CMC-GEM molecules are released, as in the case of endolysosomal vesicles. Given that amide bonds are susceptible to acid-catalyzed cleavage, the hydrolysis of CMC-GEM will result in the release of free GEM, enabling its phosphorylation and subsequent inhibition of DNA synthesis in the nucleus. This process has the potential to enhance the bioavailability of the drug compared to its conjugated form, as the high molecular weight of CMC-GEM could limit nuclear internalization.

To investigate the possible acid-catalyzed hydrolysis of CMC-GEM under simulated conditions relevant to endolysosomal vesicles, a kinetic degradation study was conducted using UV–vis spectroscopy at pH 4.5 and 37 °C (Figure 5c), aligning with the conditions used to evaluate GEM release and CaP dissolution. Additionally, comparative experiments were performed at pH 5.5 and 3.5 at 37 °C, as well as at pH 3.5 at 60 °C (Figure S7). The results revealed a progressive disappearance of absorption bands at 247 and 299 nm, corresponding to GEM conjugated to CMC, alongside the emergence of a new band at 270 nm, indicative of free GEM.

The rates of disappearance and appearance varied according to the tested conditions. Figure 5d shows the calculated rate constants for the acid-catalyzed hydrolysis of CMC-GEM, while Figure 5e presents the half-life values, assuming pseudo-first-order kinetics. The UV–vis calibration curves employed are shown in Figure S2d–f and the linear plots used for these calculations are shown in Figure S8. A clear trend was observed: the rate constant increased with decreasing pH and rising temperature, while the half-life of CMC-GEM decreased accordingly. At pH 4.5, the rate constant was determined to be 0.02056 ± 0.00141 h^–1^, with a half-life of 34 ± 2 h, suggesting that deconjugation occurs at a rate comparable to GEM release and CaP dissolution under the same acidic conditions. These findings indicate that both processes are expected to take place simultaneously.

Based on the kinetic studies of GEM release, Ca^2+^ dissolution, and CMC-GEM hydrolysis, Figure 5f illustrates the pH-responsive mechanism of GEM-loaded CaP NPs. Under acidic conditions, the dissolution of the CaP core facilitates drug release, while the hydrolysis of the CMC-GEM polymer allows free GEM bioavailability.

### 2.6 Cellular Internalization of Nanoparticles

The study of cellular internalization is essential to confirm the uptake of NPs by target cells and to evaluate the potential for enhanced delivery of CaP-GEM-FA NPs. We initially investigated the internalization of these NPs in MCF-7 cells, a FRα-overexpressing breast adenocarcinoma cell line. As shown in Figure 6, using SEM we observed that CaP-GEM-FA NPs were already present on the cell surface after just 1 h of incubation. To examine whether the particles had also been taken up by the cells, we performed freeze-fracture analysis. Interestingly, even at this early stage, some NPs were found inside the cells, indicating that internalization had already begun. At the 4- and 6-h time points, particles were still clearly visible on the surface of the cells. Overall, these observations suggest that nanoparticle-cell contact is quickly established and remains stable for hours, with both surface association and internalization occurring relatively early during exposure.

**Figure 6:**
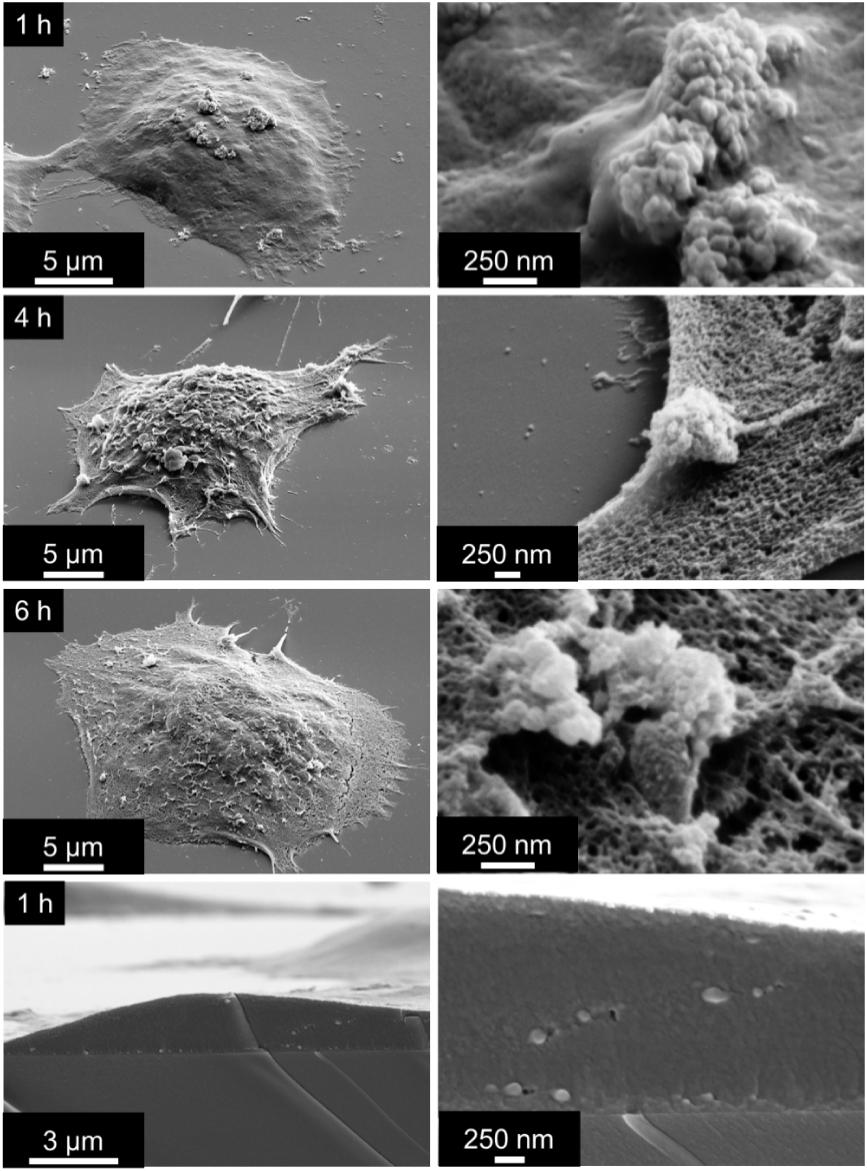
SEM images showing the interaction of CaP-GEM-FA NPs with the MCF-7 cell surface at different times. Representative images taken after 1, 4, and 6 h of incubation reveal NPs adhering to the cell membrane. After 1 h, particles are already detectable on the cell surface, and freeze-fracture analysis confirmed internalization at this early stage.

Following the confirmation that uptake of NPs begins as early as 1 h, cellular internalization was further evaluated using flow cytometry. For this purpose, CaP-GEM-BR and CaP-GEM-BR-FA NPs were used, in which the NPs were labeled with the fluorophore BR, an analog of Cy5. Figure 7a illustrates the excitation and emission spectra of these NPs in the red region, demonstrating that they exhibit similar spectral profiles with comparable intensities. This ensures that both formulations maintain equivalent fluorescent properties, which is crucial for reliable internalization studies. FRα-overexpressing MCF-7 tumorigenic cells were used alongside hMSCs, a model for healthy cells with lower FRα expression. As shown in Figure 7b, the uptake of CaP-GEM-BR-FA NPs was significantly higher than that of CaP-GEM-BR NPs in MCF-7 cells, as determined by flow cytometry after incubating with 100 μg mL^–1^ of NPs for 4 h. Specifically, MCF-7 cells internalized approximately four times more CaP-GEM-BR-FA NPs than CaP-GEM-BR NPs, with a statistically significant increase in mean fluorescence intensity (MFI) in the R2-A channel (*p* < 0.0001). Extending the incubation time to 24 h increases the cellular internalization of NPs in both groups. However, the MFI of CaP-GEM-BR-FA NPs is only twice that of CaP-GEM-BR NPs. Conversely, as shown in Figure 7c, the uptake after 4 h of incubation in hMSC is comparable between both NPs, with a slightly higher internalization observed for CaP-GEM-BR-FA NPs relative to CaP-GEM-BR NPs. Upon extending the incubation to 24 h, the cellular internalization increases for both NPs, with a more pronounced difference between the two formulations, i.e., the MFI for CaP-GEM-BR-FA NPs is 1.2 times higher than that of CaP-GEM-BR NPs.

**Figure 7:**
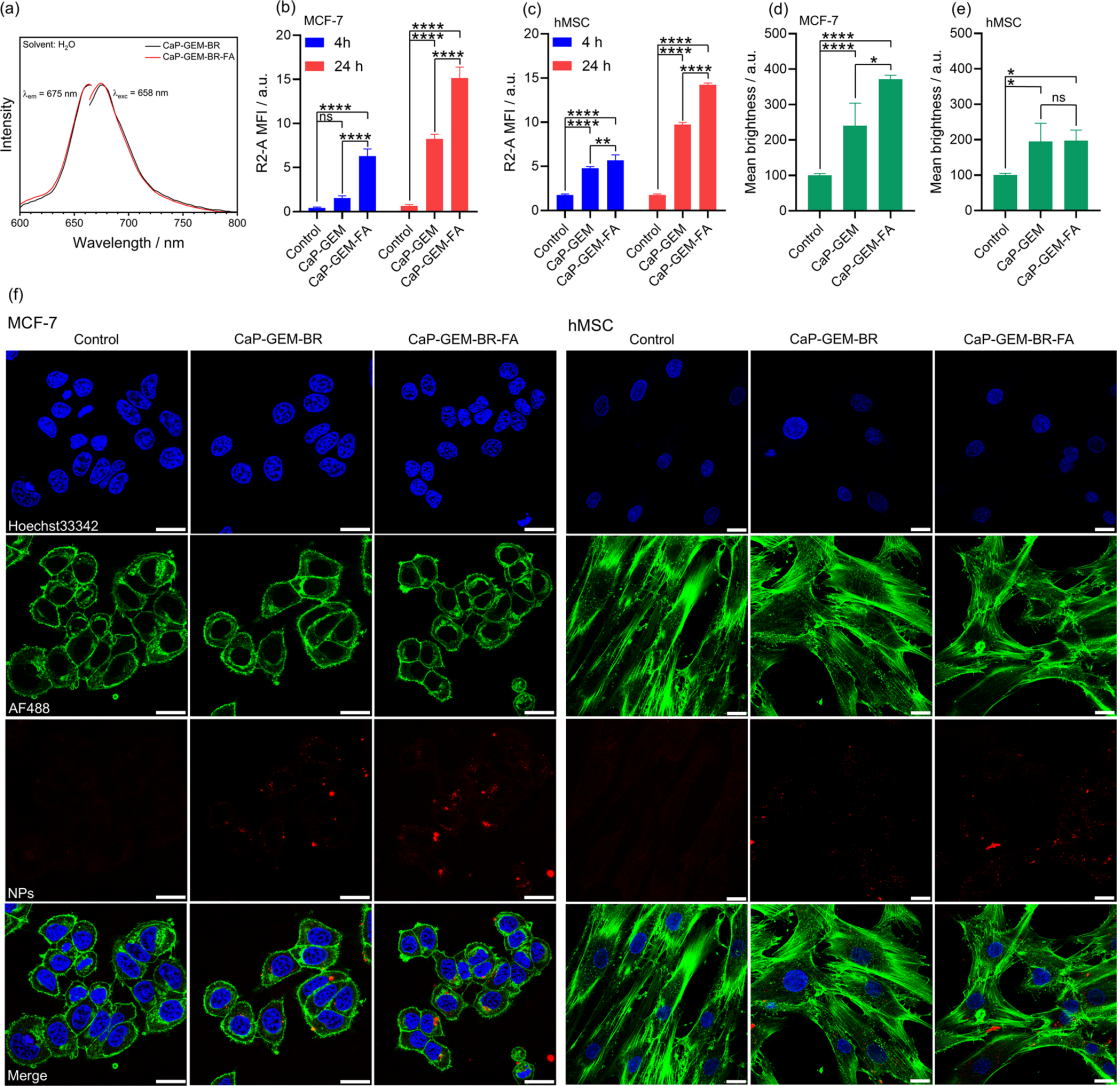
Cellular internalization of CaP NPs. (a) Excitation and emission spectra of CaP-GEM and CaP-GEM-BR-FA NPs. (b,c) Quantification of the mean fluorescence intensity (MFI) by flow cytometry after coincubation of MCF-7 and hMSC cells with 100 μg mL^–1^ of NPs for 4 or 24 h. (d,e) Fluorescence quantification based on mean pixel brightness from CLSM image analysis (NPs, λ_exc_ = 647 nm). (f) Representative CLSM images of MCF-7 and hMSC cells treated with 100 μg mL^–1^ of NPs for 4 h. Scale bar: 20 μm. Significance levels: *p* < 0.05 (*), *p* < 0.01 (**), *p* < 0.001 (***), and *p* < 0.0001 (****).

To further support the flow cytometry data, an internalization study was conducted by confocal laser scanning microscopy (CLSM) for both NPs coincubated with MCF-7 and hMSC cells for 4 h. An image processing analysis was performed to quantify the mean pixel brightness, correlating with fluorescence intensity in the red channel (λ_exc_ = 647 nm) coming from internalized NPs. The results are presented in Figure 7d,e for MCF-7 and hMSC cells, respectively. Considering the autofluorescence of MCF-7 as 100%, an increase in fluorescence intensity was observed, reaching 241% for CaP-GEM-BR NPs and 319% for CaP-GEM-BR-FA NPs. The difference in fluorescence intensity between both NPs was statistically significant (*p* < 0.05), indicating a higher uptake of FA-conjugated NPs. When analyzing the interaction with hMSC cells, a similar increase in fluorescence intensity was observed for both NPs compared to the control, reaching 195% and 197% of brightness for CaP-GEM-BR and CaP-GEM-BR-FA NPs, respectively. This suggests close uptake behavior for both NPs, consistent with the flow cytometry results. As shown in Figure 7f, the fluorescence signal from the BR fluorophore in CaP-GEM-BR and CaP-GEM-BR-FA NPs is represented by the red staining in the intracellular region of MCF-7 and hMSC cells. The NPs appeared as dispersed particles and small aggregates near the nuclear region, stained with Hoechst 33342, and colocalized with AF488-phalloidin, which labels the F-actin cytoskeleton.

Our findings suggest that distinct internalization pathways are involved depending on the presence or absence of FA on CaP NPs. For nonfunctionalized CaP NPs, such as CaP-GEM-BR, nonspecific endocytic pathways play a major on cellular internalization.^7^ However, upon FA conjugation, receptor-mediated endocytosis via the FRα receptor becomes the dominant internalization mechanism. This effect is well-documented for FA-functionalized NPs,^28^ where FRα clustering at the plasma membrane facilitates their internalization. FA conjugation on CaP NPs by CuAAC click reaction effectively reduces nonspecific cellular internalization in MCF-7 cells during short incubation periods, while this effect is nearly absent in hMSCs. However, although hMSCs are noncancerogenic, their FA receptor expression is not absent due to the high metabolic demand that is characteristic of stem cells. A study conducted by Santos et al.^44^ suggests that FA-mediated internalization can also occur in hMSCs with CaP NPs, corroborating our findings. Therefore, the greater difference in cellular uptake between FA-conjugated and nonconjugated NPs in MCF-7 cells compared to hMSCs, especially at 4 h of incubation, may be attributed to variations in FRα receptor expression density, with less nonspecific internalization occurring in MCF-7 cells than in hMSCs.

### 2.7 Cytotoxicity in Tumor Cell Lines

Figure 8 shows the MTT assay results for MCF-7 cells treated for 72 h with pure CMC (0.1 μg mL^–1^), CMC-GEM (0.1 μg mL^–1^, corresponding to 2.7 ng mL^–1^ of conjugated GEM), and free GEM (2.7 ng mL^–1^). The 2.7 ng mL^–1^ concentration was chosen for its proximity to the reported IC_50_ value of free GEM.^45^ The conjugated polymer exhibits a cytotoxicity comparable to that of the free drug at the same drug concentration, with cell viability close to 40%. In contrast, pure CMC showed no significant toxicity. These findings, together with those from the kinetic study of CMC hydrolysis, indicate that the cytotoxic effect of CMC-GEM is attributed to the release of GEM after the cellular uptake of the conjugated polymer.

**Figure 8:**
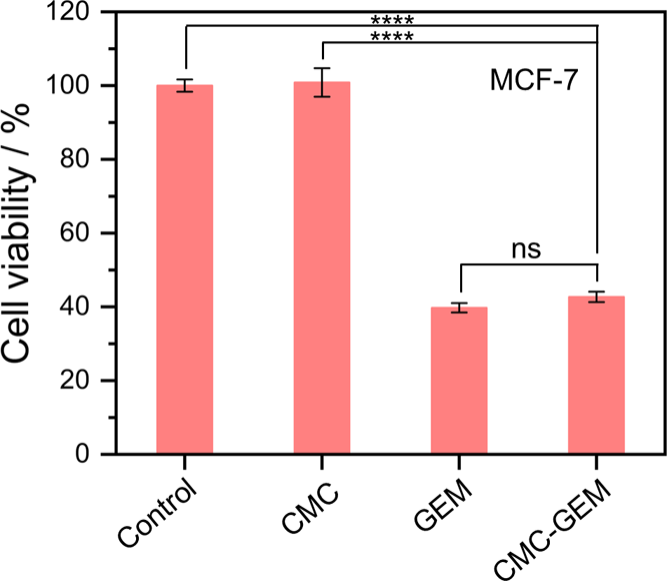
MTT assay results showing the cell viability of MCF-7 cells incubated for 72 h with CMC-GEM (2.7 ng mL^–1^ of GEM conjugated to 0.1 μg mL^–1^ of CMC), pure CMC (0.1 μg mL^–1^), and free GEM (2.7 ng mL^–1^). Significance level: *p* < 0.0001 (****).

Figure S9 shows the cytotoxicity assay for MCF-7 cells exposed to CaP, CaP-GEM, CaP-FA, and CaP-GEM-FA NPs after 24 h of incubation. The tested concentrations ranged from 0 to 100 μg mL^–1^ of CaP core, corresponding to 0 to 327 ng mL^–1^ of GEM in samples synthesized with CMC-GEM instead of pure CMC. In all cases, no significant differences are observed compared to control group. This is an expected result due to the pharmacokinetics of GEM, which usually needs 72 h for satisfactory cell inhibition.^20^ As shown in Figure 9a, it is possible to see a dose-dependent cytotoxic effect for MCF-7 cells after incubation for 72 h, with higher doses exhibiting the lower viabilities. Moreover, there are significant differences between the cytotoxicity of each sample. The full statistical analysis is presented in Figure S10.

**Figure 9:**
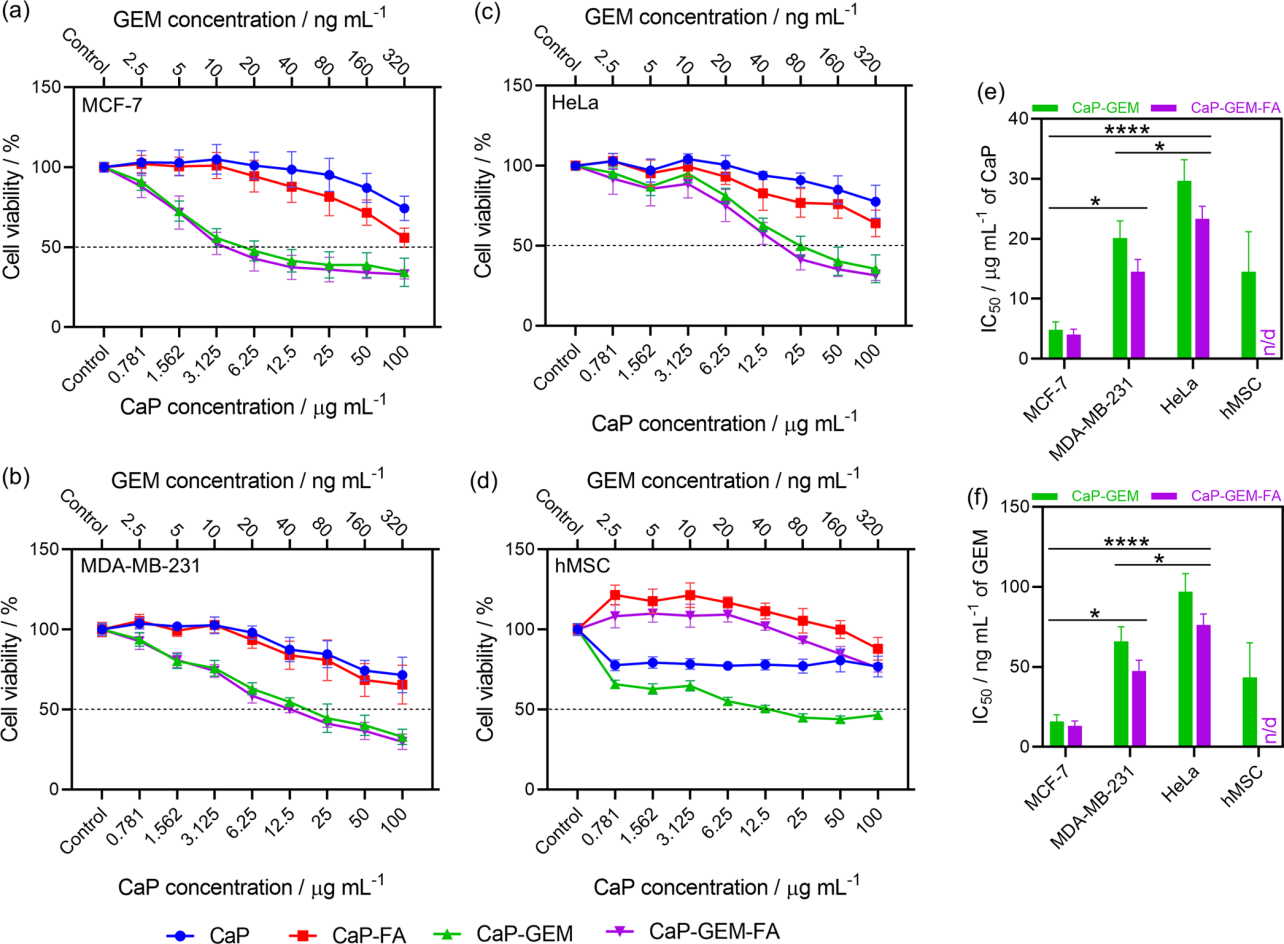
Cell viability assay with tumor and healthy cells coincubated with CaP, CaP-GEM, CaP-FA, and CaP-GEM-FA NPs for 72 h. A screening assay was conducted using different CaP core concentrations in (a) MCF-7, (b) MDA-MB-231, (c) HeLa, and (d) hMSC cells. IC_50_ values of CaP-GEM and CaP-GEM-FA NPs are shown in (e) as a function of CaP core concentration and in (f) based on the corresponding amount of loaded GEM. Significance levels: *p* < 0.05 (*), *p* < 0.01 (**), *p* < 0.001 (***), and *p* < 0.0001 (****).

CaP NPs lead to a statistically significant reduction in MCF-7 cell viability to 87% and 74% at concentrations of 50 and 100 μg mL^–1^, respectively. A similar trend is observed following FA conjugation (CaP-FA). However, the effect was more pronounced, with CaP-FA NPs reducing the cell viability to 71% and 56% at the same CaP core concentrations. It is well established that CaP NPs can induce apoptosis in cancer cells by disrupting Ca^2+^ homeostasis, as tumor cells have a reduced capacity to efflux excess Ca^2+^ ions driven by the dissolution of CaP NPs within endolysosomes.^8^ The higher cytotoxicity of FA-functionalized NPs can be attributed to their enhanced cellular uptake, as evidenced by internalization studies, possibly leading to higher intracellular Ca^2+^ accumulation and subsequent apoptotic effects. Compared to the study by Dong et al.,^46^ where 750 μg mL^–1^ of hydroxyapatite NPs reduced MCF-7 viability to 62.4% after 72 h, our CaP and CaP-FA NPs exhibited significantly high cytotoxicity at much lower concentrations. This enhanced effect is likely due to the lower crystallinity and higher solubility of our amorphous CaP NPs, as well as the improved cellular internalization of the CaP NPs functionalized with FA.

The NPs containing GEM, both CaP-GEM and CaP-GEM-FA, exhibit significantly higher cytotoxicity than the NPs without GEM, with a more pronounced and dose-dependent response. Statistically significant differences are observed from the concentration of 1.562 μg mL^–1^ of the CaP core, corresponding to ∼ 5 ng mL^–1^ of loaded GEM, with similar cell viabilities of 71%. The IC_50_ values for CaP-GEM and CaP-GEM-FA NPs are presented in Figure 9e,f, showing values of 4.83 μg mL^–1^ (15.8 ng mL^–1^ of GEM) and 4.03 μg mL^–1^ (13.2 ng mL^–1^ of GEM), respectively. The lowest cell viability was observed at 100 μg mL^–1^ of NPs (∼360 ng mL^–1^ of GEM), with values close to 33% for both NPs. Although no statistically significant differences in cytotoxicity were detected after FA functionalization, when considered together with the internalization assays, these results confirm that the CaP-GEM-FA NPs retain high cytotoxic effects comparable to CaP-GEM NPs while exhibiting enhanced uptake in FRα-overexpressing cells.

The NPs were also tested against MDA-MB-231 and HeLa cells (Figure 9b,c), showing similar dose-dependent profiles for CaP, CaP-FA, CaP-GEM, and CaP-GEM-FA NPs, reinforcing the results obtained with MCF-7 cells. The lowest cell viability was observed at 100 μg mL^–1^ (∼360 ng mL^–1^ of GEM) of CaP-GEM and CaP-GEM-FA, with values of respectively 33% and 29% for MDA-MB-231 and 36% and 31% for HeLa. The main difference lies in the higher IC_50_ values for GEM-loaded NPs. As illustrated in Figure 9e,f, for MDA-MB-231, the IC_50_ values for CaP-GEM and CaP-GEM-FA are 20.1 μg mL^–1^ (65.9 ng mL^–1^ of GEM) and 14.5 μg mL^–1^ (47.6 ng mL^–1^ of GEM), respectively. For HeLa cells, the IC_50_ values are 29.7 μg mL^–1^ (97.1 ng mL^–1^ of GEM) and 23.3 μg mL^–1^ (76.2 ng mL^–1^ of GEM). This trend of higher IC_50_ values in MDA-MB-231 and HeLa cells compared to MCF-7 aligns with literature,^45^ as these cell lines are known to be more aggressive and highly proliferative.

### 2.8 Cytotoxicity in Mesenchymal Stem Cells

Figure 9d shows the cell viability data for the NPs on hMSC cells. Compared to tumor cell lines, the viability profiles differ significantly for all NPs. In the case of CaP NPs, cell viability remained around 78% across all tested concentrations. The absence of a dose-dependent effect suggests that hMSC cells can tolerate a certain level of CaP NP exposure without additional cytotoxic effects at higher concentrations. However, when FA is conjugated to the NP surface, viability values exceeding 100% are observed at CaP concentrations up to 50 μg mL^–1^. At 0.781 μg mL^–1^ of CaP-FA, viability reached 125%, gradually decreasing to 100% at 50 μg mL^–1^ and 88% at 100 μg mL^–1^. The differences compared to the control were statistically significant up to 12.5 μg mL^–1^.

The MTT assay correlates mitochondrial activity with cell viability, with values exceeding 100% indicating stimulated mitochondrial function, which may reflect enhanced cellular proliferation and metabolic activity. In this context, FA conjugation to CaP NPs may promote hMSC proliferation by supporting DNA synthesis. This occurs through the role of FA in purine and pyrimidine nucleotide production, which is essential for DNA replication and cell cycle progression.^47^ Additionally, FA enhances energy metabolism by contributing to NADPH synthesis, supporting anabolic processes and redox homeostasis,^44^ thereby improving cell division and viability. However, the observed decrease in viability from 125% to 88% as CaP-FA concentrations increase may reflect a balance between the beneficial effects of FA and potential cellular stress induced by higher NPs and FA concentrations.

In contrast, CaP-GEM demonstrates a clear toxicity effect, with cell viability dropping to 65.8% at 0.781 μg mL^–1^ and 46% at 100 μg mL^–1^, which reflects the cytotoxicity of GEM release from the NPs. The IC_50_ of CaP-GEM (14.5 μg mL^–1^ CaP core, or 43.5 ng mL^–1^ GEM) indicates that the cytotoxic effects of GEM are significant even at relatively low concentrations. When FA is conjugated to the NPs (CaP-GEM-FA), the behavior observed is similar to that of CaP-FA, with significantly higher cell viability in comparison to CaP-GEM. Furthermore, the CaP-GEM-FA sample shows a general trend of lower viability than CaP-FA, which is expected due to the presence of GEM. However, at various concentrations, the differences between CaP-GEM-FA and CaP-FA are not statistically significant. This suggests that the role of FA in supporting nucleotide biosynthesis and DNA replication counteracts the toxicity associated with the DNA damage by GEM molecules.

Overall, our results demonstrate enhanced receptor-mediated internalization and pronounced dose-dependent cytotoxicity of the CaP-CMC-GEM/SiO_2_–FA nanocarrier (abbreviated as CaP-GEM-FA) toward FRα-overexpressing cancer cells, confirming its selective antitumor effect while minimizing toxicity to healthy cells. To achieve this, the dual pH-responsive nanosystem is constructed through the integration of a CaP core, a CMC intermediate layer, and an outer SiO_2_ shell obtained by a simple continuous aqueous precipitation followed by a modified Stöber process, yielding stable NPs with minimized long-term toxicity concerns. Its functionality arises from the abundance of reactive groups, such as the carboxyl groups on CMC, which enable an intracellularly cleavable amide bond with GEM, and the silanol groups on SiO_2_, which can be further modified with azide moieties to allow a CuAAC click reaction with FA-PEG3-propargyl. The later ensures efficient and stable surface targeting for potential application in cancer therapy.

Although effective GEM loading in CaP NPs has been achieved in previous studies, as in Lipid/Calcium/Phosphate (LCP) systems that immobilize phosphorylated GEM onto the CaP core and are coated with lipid bilayers bearing targeting ligands (e.g., anisamide or cyclic RGD peptides), their fabrication relies on a reverse microemulsion process that is complex and often low yielding at a small scale.^21–23,48^ In another approach, GEM was conjugated to mPEG-*b*-PLG via amide bond formation and used to precipitate CaP NPs, though lacking targeting ligands and therefore showing limited selectivity toward cancer cells.^24^ A similar limitation is observed for the hydroxyapatite-PVA core–shell nanocarrier conjugated with methotrexate and physically loaded with GEM, in which targeting moieties were not incorporated.^49^

Beyond CaP-based carriers, GEM has also been delivered using liposomes, polymeric NPs, mesoporous silica, iron oxide, gold, and other inorganic systems.^50^ However, liposomes often suffer from high manufacturing costs, limited scalability, and stability issues that lead to premature drug leakage, particularly problematic since GEM can diffuse through the liposomal bilayer.^22,51^ In addition, polymeric carriers may exhibit systemic toxicity from degradation products, while some nonbiodegradable inorganic NPs raise concerns about metabolism, excretion, and long-term safety *in vivo*.^52^ In this context, natural biomineral-based nanostructured materials, such as our CaP NPs, stand out as ideal biocompatible, biodegradable and pH-responsive drug-delivery platforms.^8^ These considerations highlight the potential of the developed CaP-CMC-GEM/SiO_2_–FA nanocarrier as a versatile and promising platform for targeted GEM delivery and as a generalizable system for the conjugation of other antineoplastic drugs and targeting moieties.

## 3 Conclusions

We demonstrated that CaP NPs stabilized with CMC and coated with a SiO_2_ layer can be employed for the delivery of GEM. Prior to CaP NPs precipitation, GEM was covalently conjugated to CMC via a novel amide bond formation, achieving a substantial degree of substitution (2.74 wt %). This conjugated polymer not only stabilizes the NPs in suspension, maintaining controlled size distributions, but also enables covalent drug attachment, effectively overcoming the limitations of poor electrostatic interactions, which restrict GEM loading capacity and may lead to premature release. Our findings emphasize the pH-responsive nature of these NPs, where GEM release remains below 30% at physiological pH (7.4) but reaches complete release (100%) under endolysosomal conditions (pH 4.5). This release profile is coupled with a concurrent Ca^2+^ release, supporting a controlled degradation mechanism driven by the dissolution of the CaP core. Additionally, the acid-catalyzed hydrolysis of CMC-GEM further ensures intracellular bioavailability of free GEM, establishing a dual pH-responsive system wherein drug release is governed both by CaP dissolution and by the cleavage of the polymer-drug conjugate.

FA molecules were efficiently conjugated to the SiO_2_ layer of CaP and CaP-GEM NPs via CuAAC click chemistry, forming highly stable covalent bonds. Internalization assays in MCF-7 cells confirmed that FA significantly enhances the uptake of NPs within the first 4 h of incubation, consistent with the high expression of FRα in these cells. In hMSC cells, the internalization rates of FA-conjugated and nonconjugated NPs were comparable at short periods, due to the balance between receptor-mediated and nonspecific endocytosis, as FA receptor expression is lower in these cells.

Cytotoxicity studies revealed that CaP-GEM NPs exhibited strong cytotoxic effects after 72 h against FRα-overexpressing cancer cell lines (MCF-7, MDA-MB-231, and HeLa), as well as on hMSCs, with IC_50_ values ranging from 4.83 μg mL^–1^ to 29.7 μg mL^–1^ of NPs, corresponding to 15.8 ng mL^–1^ to 97.1 ng mL^–1^ of conjugated GEM. On the other hand, CaP-FA demonstrated toxicity toward cancer cells at higher concentrations (50–100 μg mL^–1^) due to increased internalization of NPs via FA receptor-mediated endocytosis. Instead of inducing cytotoxic effects, CaP-FA NPs enhanced mitochondrial activity of hMSC cells, possibly by facilitating FA-dependent metabolic pathways, such as DNA synthesis and methylation processes, which are crucial for cell proliferation and function. Interestingly, FA-conjugated NPs carrying GEM maintained high cytotoxicity in cancer cells, while in hMSCs, their behavior closely resembled that of FA-functionalized NPs without GEM. This suggests that the beneficial effects of FA may balance the DNA-damaging and antiproliferative effects of GEM in healthy cells. These findings underscore the potential of this dual pH-responsive nanoplatform for targeted cancer therapy.

## 4 Materials and Methods

### 4.1 Reagents

The following reagents were used: carboxymethylcellulose sodium salt (CMC; DS 0.7, *M*_w_ ∼ 90 kDa, Sigma-Aldrich), 1-ethyl-3-(3-(dimethylamino)­propyl)­carbodiimide hydrochloride (EDC; ≥99%, Carl Roth), *N*-hydroxysuccinimide (NHS; 98%, Sigma-Aldrich), gemcitabine hydrochloride (GEM; >98%, TCI), calcium lactate pentahydrate (USP Reference Standard, Sigma-Aldrich), ammonium hydrogen phosphate ((NH_4_)_2_HPO_4_; ≥98%, VWR Life Science), tetraethyl orthosilicate (TEOS; 98%, Merck), CMC-BR (label degree 1:66, *M*_w_ ∼ 200 kDa, Surflay Nanotec), (3-azidopropyl)­triethoxysilane (97%, SelectLab Chemicals), (*S*)-17-(4-(((2-amino-4-oxo-3,4-dihydropteridin-6-yl)­methyl)­amino)­benzamido)-14-oxo-4,7,10-trioxa-13-azaoctadec-1-yn-18-oic acid (Folate-PEG3-propargyl; 97%, AmBeed), copper sulfate pentahydrate (CuSO_4_·5H_2_O; 99%, AppliChem), tris­(3-hydroxypropyl-triazolylmethyl)­amine (THPTA; 95%, Sigma-Aldrich), aminoguanidine hydrogen carbonate (98+%, Alfa Aesar), sodium ascorbate (≥99%, Sigma-Aldrich), potassium bromide (KBr; Sigma-Aldrich), dimethylformamide (DMF; Fisher Scientific), dimethyl sulfoxide (DMSO; Fisher Scientific), absolute ethanol (Fisher Scientific), ammonia solution (30%, Carl Roth), sodium hydroxide (NaOH; 0.1 M, Bernd Kraft), glacial acetic acid (Fisher Scientific), deuterium oxide (D_2_O; 99.9%, Deutero), chloride acid solution (HCl; 1 M, Bernd Kraft), Dulbecco’s Modified Eagle’s Medium (DMEM; Gibco, Thermo Fisher Scientific), fetal bovine serum (FBS; Gibco, Thermo Fisher Scientific), RPMI 1640 (Gibco, Thermo Fisher Scientific), penicillin (Gibco, Thermo Fisher Scientific), streptomycin (Gibco, Thermo Fisher Scientific), GlutaMAX (Gibco, Thermo Fisher Scientific), Dulbecco’s phosphate-buffered saline (DPBS; Gibco, Thermo Fisher Scientific), TrypLE Express Enzyme (Gibco, Thermo Fisher Scientific), hexamethyldisilazane (HMDS; Sigma-Aldrich), MACSQuant running buffer (Miltenyi Biotec), paraformaldehyde (PFA; p.a., Merck), AF488 phalloidin conjugate (AAT Bioquest, Biomol), Hoechst 33342 (Life Technologies), and 3-(4,5-dimethylthiazol-2-yl)-2,5-diphenyltetrazolium bromide (MTT; Invitrogen, Thermo Fisher Scientific). For all experimental procedure, ultrapure water (Purelab Ultra, Elga, Germany) was used.

### 4.2 GEM Conjugation to CMC Polymer

The CMC-GEM precursor was prepared prior to the synthesis of CaP NPs by promoting the formation of an amide bond between the activated carboxyl groups of CMC and the primary amino groups of GEM. Initially, 20 mg of CMC was dissolved at 55 °C in 10 mL of a solvent mixture composed of DMF and DMSO in a 3:1 ratio, with 5% (*v/v*) ultrapure water. The solution was continuously stirred for 5 min and then cooled to room temperature. Then, EDC, NHS, and GEM were added to the CMC solution in a 1:2:1 molar ratio, yielding final concentrations of 6.67 mM, 13.29 mM, and 6.67 mM, respectively. The reaction mixture was stirred at 450 rpm for 24 h and subsequently purified using five 10 kDa MWCO spin filters (Amicon Ultra-15, Merck Millipore) at 4000 rpm for 25 min, repeated 8 times with ultrapure water (10 mL per tube). The final product was either diluted in 10 mL of H_2_O to prepare a CMC-GEM solution at 2 mg mL^–1^ based on the CMC content and stored at −80 °C or freeze-dried using a Christ Alpha 2–4 LSC instrument (Martin Christ, Germany) to obtain the white CMC-GEM solid.

### 4.3 Synthesis of GEM-Loaded CaP NPs

The synthesis procedure to obtain the CaP-CMC-GEM/SiO_2_ NPs (abbreviated as CaP-GEM) follows the approach outlined by Kozlova et al.,^16^ with some modifications. Aqueous solutions of calcium lactate pentahydrate (18 mM, pH 10), (NH_4_)_2_HPO_4_ (10.8 mM, pH 10), and CMC-GEM (2 mg mL^–1^) were simultaneously pumped at room temperature for 1 min using two peristaltic pumps under stirring at 850 rpm. The pumping occurred in a volume ratio of 5 mL:5 mL:7 mL into a glass vessel containing 20 mL of ultrapure water. The resulting suspension of NPs was stirred for an additional 20 min to achieve a homogeneous suspension.

The silica coating on the NPs was achieved using a methodology based on the Stöber method^53^ and adapted by Kozlova et al.^16^ In this methodology, 10 mL of the NPs obtained in the former step were added to a mixture of 40 mL absolute ethanol, 50 μL of TEOS and 100 μL of 7.8 wt % aqueous ammonia solution. The reaction mixture was stirred for 16 h at room temperature. Then, the NPs were isolated by centrifugation at 14000 rpm per 30 min and redispersed in the original volume of water (10 mL) with an ultrasound bath. A pure CaP-CMC/SiO_2_ sample (abbreviated as CaP) without GEM conjugation was synthesized using the same protocol, replacing CMC-GEM with CMC.

### 4.4 Synthesis of Fluorescent Dye-Labeled CaP NPs

The CaP NPs were labeled with the BR fluorophore (λ_exc_ = 655 nm, λ_em_ = 674 nm), a more photostable Cy5 analog, which had been preconjugated to the CMC polymer by the supplier. The synthesis procedure is similar to that described in Section 4.3, except that CMC-BR was mixed with CMC-GEM aqueous solution at final concentrations of 0.2 mg mL^–1^ and 2 mg mL^–1^, respectively, to prepare the CaP-CMC-GEM-BR/SiO_2_ NPs (abbreviated as CaP-GEM-BR).

### 4.5 FA-Conjugation by CuAAC Click Reaction

The conjugation of FA to the CaP, CaP-GEM, and CaP-GEM-BR NPs was performed using a copper­(I)-catalyzed azide–alkyne cycloaddition (CuAAC) click reaction. Initially, to produce the azide-terminated NPs, 10 mL of silica-modified NPs were mixed with a solution containing 40 mL of ethanol, 50 μL of (3-azidopropyl)­triethoxysilane, and 50 μL of a 7.8 wt % aqueous ammonia solution. The reaction mixtures were stirred at room temperature for 8 h. Subsequently, a centrifugation step at 14000 rpm for 30 min was performed to isolate the NPs, which were then resuspended in 5 mL of ultrapure water using an ultrasound bath.

In the next step, 1.0 mL of the azide-terminated NPs were mixed, in the following order, with 1.6 μL of NaOH 0.1 M, 100 μL of folate-PEG3-propargyl at 1 mg mL^–1^ in DMSO, 8 μL of an aqueous solution containing CuSO_4_ (40 μM, 1000 μM, or 5000 μM) and THPTA (9.66 mM), 83 μL of an aqueous solution of aminoguanidine (7.35 mM), and, finally, 83 μL of an aqueous solution of ascorbate (100 mM) to initiate the reaction. The mixture was stirred at 650 rpm for 1 h at room temperature. Subsequently, the NPs were isolated by centrifugation at 14000 rpm for 30 min, followed by washing with 1 mL of water. The NPs were then redispersed in 1 mL of water using an ultrasound bath. This procedure resulted in the CaP-FA, CaP-GEM-FA, and CaP-GEM-BR-FA NPs.

### 4.6 CMC-GEM Characterization

The absorption spectra of CMC, GEM, and CMC-GEM were obtained by UV–vis spectroscopy using a Genesis 50 instrument (Thermo Scientific, USA) with quartz glass cuvettes, in the range of 200 to 400 nm in water. The concentration of conjugated GEM in the CMC-GEM polymer was determined after complete hydrolysis of the newly formed amide bond, performed at 60 °C and pH 3.5 in acetate buffer for 24 h at a CMC-GEM concentration of 0.5 mg mL^–1^. A calibration curve (Figure S2a), constructed according to Lambert–Beer’s law for free GEM at pH 3.5 in acetate buffer, was used to quantify the concentration of free GEM after hydrolysis, which was then correlated to the amount of conjugated GEM. The detailed calculations can be found in the SI file. Nuclear magnetic resonance (NMR) spectra were recorded with Bruker Avance Neo 400 MHz (Bruker, Germany) and Avance III 600 MHz (Bruker, Germany) spectrometers in D_2_O. Attenuated Total Reflectance (ATR) Fourier Transform Infrared (FTIR) spectra were recorded using an Alpha spectrometer (Bruker, Germany) within the wavenumber range of 4000 cm^–1^ to 400 cm^–1^, with a resolution of 2 cm^–1^ and 24 scans. The spectra were measured in ATR mode and automatically converted to transmittance using OPUS software (v7.0). Elemental analysis of the CMC-GEM compound (∼5 mg) was performed using an EA3100 Elemental Analyzer (Euro Vector, Italy) to determine the percentages of C, H, N, and O.

### 4.7 Characterization of CaP NPs

The calcium concentration in the NP suspensions was determined using atomic absorption spectroscopy (AAS) with an M-Series AA spectrometer (Thermo Electron Corporation, USA). For the analysis, 50 μL of the suspensions were mixed with 50 μL of HCl 1 M and diluted to a final volume of 5 mL with water. The quantification of conjugated-GEM on the NPs was performed using a direct UV–vis method. A suspension containing GEM-loaded CaP NPs, quantified as 220 μg based on AAS results, was centrifuged at 14000 rpm for 30 min to produce a pellet. The resulting pellet was dissolved in 400 μL of an aqueous solution of HCl at 0.125 M. The UV–vis spectrum was then recorded in the range of 200–400 nm, and the absorbance at 247 nm was used to quantify GEM based on a calibration curve established for CMC-GEM (Figure S2b). From this data, the concentration of CMC on the NPs was also determined. In the case of conjugated-FA NPs, a pellet corresponding to 85 μg of NPs, as determined by AAS, was dissolved in 300 μL of an aqueous HCl solution at 0.166 M. The UV–vis spectrum was recorded in the range of 200–500 nm, with absorbance monitored at 300 nm and compared to a calibration curve established for folate-PEG3-propargyl (Figure S3b). All the reported values and their corresponding standard deviations refer to data obtained from quantifications in at least three independent syntheses. The detailed calculations can be found in the SI file.

FTIR measurements were performed using a Vertex 70 spectrometer (Bruker, USA) on pellets prepared with 1 mg of freeze-dried NPs and 200 mg of KBr, covering the 4000–400 cm^–1^ range in transmittance mode, with a resolution of 4 cm^–1^ and 32 scans. The hydrodynamic size by means of Z-average (Z-Avg), polydispersity index (PDI) and zeta potential of the NPs were determined in a Nano ZS ZEN 3600 instrument (Malvern Instruments, UK) using laser wavelength of 633 nm at 25 °C with a DTS1070 disposable folded capillary cuvette. The morphology and CaP core sizes of the NPs were analyzed by Scanning Electron Microscopy (SEM) using an Apreo S LoVac microscope (Thermo Fisher Scientific, USA). To prepare the samples, 5 μL aliquots of the NP suspensions were deposited onto sample holders, air-dried, and sputter-coated with gold/palladium. The average particle diameters were calculated by analyzing 100 NPs. The morphological features of the NPs were further examined by Transmission Electron Microscopy (TEM) using a FEI TECNAI G^2^ F20 microscope (Thermo Fisher Scientific, USA). The samples were prepared by depositing 10 μL aliquots of the NP suspensions onto Formvar/carbon-coated copper grids.

### 4.8 GEM and Ca^2+^ Release Profiles

The release profiles of GEM and Ca^2+^ were evaluated using 6 mg of GEM-loaded CaP NPs (equivalent to 19.6 μg of conjugated GEM) dispersed in 1 mL of HEPES or acetate buffer at pH 7.4 and 4.5, respectively. These pH values were chosen to mimic the physiological conditions of the bloodstream and endolysosomes. The suspensions were stirred at 120 rpm at 37 °C, and at predetermined time intervals, they were centrifuged at 14000 rpm for 30 min. The supernatants were collected, and 1 mL of fresh buffer was added as the replacement. The collected supernatants were then incubated at 80 °C for 24–48 h to ensure complete hydrolysis of CMC-GEM. Released GEM concentration in the supernatant was quantified by UV–vis spectroscopy at 270 nm, corresponding to the primary absorption peak of free GEM, while Ca^2+^ concentrations were determined by AAS.

### 4.9 CMC-GEM Acidic Hydrolysis

A kinetic study of the CMC-GEM acidic hydrolysis was conducted using 5 mg of CMC-GEM (equivalent to ∼137 μg of conjugated GEM) dissolved in 10 mL of acetate buffer at pH 3.5, 4.5, or 5.5. The samples were maintained under gentle stirring at 120 rpm at 37 or 60 °C. At predetermined intervals, 400 μL aliquots were collected, and their absorption spectra were recorded by UV–vis spectroscopy in the range of 200–400 nm. Over time, the absorption bands corresponding to the conjugated form of GEM decreased (247 and 299 nm), while those of the free GEM emerged (268–272 nm), indicating hydrolysis progression. Subsequently, the pseudo-first order rate constant at acid conditions (*k*′) and the corresponding half-life times (*t*_1/2_) of the hydrolysis reaction were calculated by considering the following equations:

1 $$v=k^{\prime }\left[\mathrm{Conjugated}\;\mathrm{GEM}\right]$$

2 $$k^{\prime }=k\left[H_{3}O^{+}\right]$$

3 $$t_{1/2}=\frac{\ln2}{k^{\prime }}$$

The *k*′ values were determined by monitoring the extinction of conjugated GEM, with its concentration over time calculated using calibration curves established for CMC-GEM at 247 nm in acetate buffer at pH 3.5, 4.5, and 5.5 (Figure S2d–f).

### 4.10 Stability and Protein Corona Formation

CaP-GEM-FA NPs were dispersed at a concentration of 100 μg mL^–1^ in 1 mL of H_2_O or 1 mL of a 50:50 mixture of water and DMEM supplemented with 10% FBS. The samples were kept under constant agitation at 120 rpm and 37 °C. At predetermined time intervals, aliquots were directly analyzed at 37 °C for hydrodynamic size (Z-Avg), PDI, and zeta potential. At the end of the experiment, the dried samples were analyzed by ATR-FTIR spectroscopy.

### 4.11 Cell Culture

In this study, breast adenocarcinoma cells (MCF-7 and MDA-MB-231), cervical adenocarcinoma cells (HeLa), and human mesenchymal stem cells (hMSC) were cultured in T75 cell culture flasks at 37 °C with a 5% CO_2_ atmosphere for subsequent *in vitro* assays. The cell culture media used were RPMI 1640 for MCF-7 cells and DMEM for MDA-MB-231, HeLa, and hMSC cells, both supplemented with 10% FBS, 100 U mL^–1^ penicillin, and 100 U mL^–1^ streptomycin. Additionally, DMEM was further supplemented with 1% GlutaMAX.

### 4.12 Cellular Internalization in Target Cells

For SEM analysis, MCF-7 cells were seeded onto coated glass microscopy slides in 12-well plates (3.0 × 10^4^ cells per well). After 24 h of incubation in the media, the cells were incubated with CaP-GEM-FA NPs (100 μg mL^–1^) for 1, 4, and 6 h. Untreated cells served as negative control. For fixation, the cells were treated for 15 min at room temperature with 3.7% PFA solution, washed three times with DPBS and dehydrated with an ascending ethanol row (20%, 40%, 60%, 80% and 96%) for 5 min for each sequence. After the final ethanol step, the samples were immersed in 100% HMDS for 3 min, then dried by HMDS evaporation at room temperature and subsequently coated with gold/palladium. Cross sections were prepared by freeze-fracturing the glass microscopy slides after shock immersion in liquid nitrogen.

The quantification of cellular internalization was performed using a MACSQuant Analyzer 16 flow cytometer (Miltenyi Biotec, Germany). For the analysis, MCF-7 and hMSC cells were seeded at a density of 3.0 × 10^4^ cells per well in 12-well plates with 1 mL of complete medium. After 24 h of incubation at 37 °C under 5% CO_2_, the culture medium in each well was replaced with 1 mL of NPs conjugated with the BR fluorophore, dispersed in complete medium at a concentration of 100 μg mL^–1^, and further incubated for 4 or 24 h. Untreated cells, in which the medium was replaced with 1 mL of fresh complete medium, served as the negative control. After incubation, cells were washed thrice with 1 mL of DPBS, detached using 500 μL of TrypLE Express, centrifuged at 300 × g for 5 min, and resuspended in 500 μL of running buffer for analysis. Fluorescence data were acquired by recording 15000 events within the single cells gate and analyzed using FlowJo software.

Cellular internalization was also studied using confocal laser scanning microscopy (CLSM). MCF-7 and hMSC cells were seeded in 8-well plates at a density of 1.25 × 10^4^ cells per well with 250 μL of complete medium and incubated for 24 h at 37 °C under 5% of CO_2_ to promote cell adhesion and recovery. The culture medium was then replaced with 250 μL of complete medium containing BR-labeled NPs at a concentration of 100 μg mL^–1^, while untreated cells served as the negative control by replacing with 250 μL of fresh complete medium. Cells were further incubated for 4 h, washed thrice with 250 μL of DPBS, and fixed with 150 μL of 3.7% PFA at room temperature for 15 min. After fixation, cells were washed twice with 1 mL of DPBS, followed by F-actin labeling with an AF488 phalloidin. For this, cells were incubated for 20 min at 37 °C (5% CO_2_) with 230 μL of a working solution prepared by diluting 5 μL of the 300× stock solution in 2 mL of DPBS. The wells were then washed twice with 250 μL of DPBS before staining the nucleus Hoechst 33342. The staining solution was prepared by diluting 4 μL of the stock solution in 2 mL of DPBS, and the cells were incubated with 230 μL of this solution for 15 min at room temperature. After staining, the wells were washed twice with 250 μL of DPBS, and 250 μL of DPBS was added before storing the plates at 4 °C until analysis. Cells were imaged using a TCS SP8X Falcon microscope (Leica, Germany) with laser wavelengths of 405 nm (Hoechst33342), 488 nm (AF488 phalloidin), and 630 nm (NPs) using a HC PL APO CS2 63×/1.40 oil immersion lens. CLSM images of MCF-7 and hMSC control cells, as well as cells coincubated with NPs, were acquired under identical conditions to differentiate cellular autofluorescence from the fluorescence emission of internalized CaP-GEM-BR and CaP-GEM-BR-FA NPs. Image analysis was performed using the HSB color model, which represents each pixel by three values: Hue, Saturation, and Brightness. Since brightness correlates with the light emission captured by the microscope detector, the average pixel brightness in the images (*n* = 3) was quantified using a Python script with the OpenCV library.^42^

### 4.13 Cytotoxicity by MTT

Initially, the cytotoxicity of the CMC-GEM conjugated polymer was assessed using the MTT assay against MCF-7 cells. Cells were seeded in 96-well plates at a density of 3 × 10^3^ cells per well in 100 μL of complete culture medium. After 24 h, the medium was replaced with 100 μL of fresh complete medium containing dispersed CMC-GEM at 100 μg mL^–1^ of CMC, corresponding to 2.7 ng mL^–1^ of conjugated GEM. For comparison, CMC at 100 μg mL^–1^ and free GEM at 2.7 ng mL^–1^ were also tested. The cells were then incubated for 72 h. After the incubation, the medium was removed, and 100 μL of MTT solution (0.5 mg mL^–1^) was added to each well. The plates were incubated for 1 h, followed by the removal of all liquid and the addition of 100 μL of DMSO. Absorbance was measured at 570 nm using a Multiskan FC microplate photometer (Thermo Fisher Scientific, USA). Cell viability was determined by comparing the absorbance values of the treatment groups with those of the control group.

The MTT assay was performed to evaluate the cytotoxic effects of CaP, CaP-GEM, CaP-FA, and CaP-GEM-FA NPs on MCF-7, MDA-MB-231, HeLa, and hMSC cell lines. Cells were seeded in 96-well plates at a density of 3 × 10^3^ cells per well in 100 μL of complete culture medium. After 24 h, the medium was replaced with 100 μL of fresh complete medium containing dispersed NPs at concentrations of 0, 0.781, 1.562, 3.125, 6.25, 12.5, 25, 50, and 100 μg mL^–1^. The cells were then incubated for 24 or 72 h. Following incubation, the NP-containing medium was removed, and 100 μL of MTT solution (0.5 mg mL^–1^) was added to each well. After 1 h of incubation, the solution was discarded, and 100 μL of DMSO was added. Absorbance was measured at 570 nm, and cell viability was determined by comparing the absorbance values of the treatment groups with those of the control group.

### 4.14 Statistical Analysis

All cell experiments were performed in three independent biological replicates. Data were analyzed using two-way analysis of variance (ANOVA) followed by Tukey’s multiple comparisons test using GraphPad Prism 8. Significance levels were set at *p* < 0.05 (*), *p* < 0.01 (**), *p* < 0.001 (***), and *p* < 0.0001 (****).

## Supplementary Material



## Notes

Comprehensive characterization data including FTIR, ^1^H NMR, and ^19^F NMR spectra of CMC, GEM, and CMC-GEM; detailed procedures and calculations for CMC-GEM quantification, nanoparticle concentration, nanoparticle number, GEM loading, CMC loading, and folic-acid conjugation efficiency; UV–vis calibration curves for GEM, CMC-GEM, and folate-PEG3-propargyl; DLS size distribution profiles; EDS spectrum of CaP-GEM-FA nanoparticles; TEM micrographs of all NPs; UV–vis spectra associated with the acid-catalyzed hydrolysis of CMC-GEM; linear fits used to determine rate constants and half-lives for CMC hydrolysis; and MTT assays performed after 24 and 72 h (PDF)
