# Supplementary material for: Dual pH-Responsive Calcium Phosphate Nanoparticles Conjugated with Folate by CuAAC Click Chemistry for Targeted Gemcitabine Delivery to Cancer Cells
Source: ACS Appl Bio Mater. 2025 Dec 16;9(1):137–52. doi: 10.1021/acsabm.5c01683 (PMC12776572; doi:10.1021/acsabm.5c01683)
Supplement: Supplementary file 1 [file mt5c01683_si_001.pdf]

SUPPORTING INFORMATION

Dual pH-responsive calcium phosphate nanoparticles conjugated with folate by CuAAC click chemistry for targeted gemcitabine delivery to cancer cells

Thales R. Machado,[†,‡,] Aileen Winter,[†] Kateryna Loza,[†] Kathrin Kostka,[†] Valtencir Zucolotto,[‡] Matthias Epple[†]*

[†]Inorganic Chemistry, Centre for Nanointegration Duisburg-Essen (CENIDE), University of Duisburg-Essen, 45117 Essen, Germany.

[‡]GNANO – Nanomedicine and Nanotoxicology Group, São Carlos Institute of Physics, University of São Paulo, 13566-590 São Carlos, SP, Brazil.

*Corresponding author: Thales R. Machado (trmachado@ifsc.usp.br)

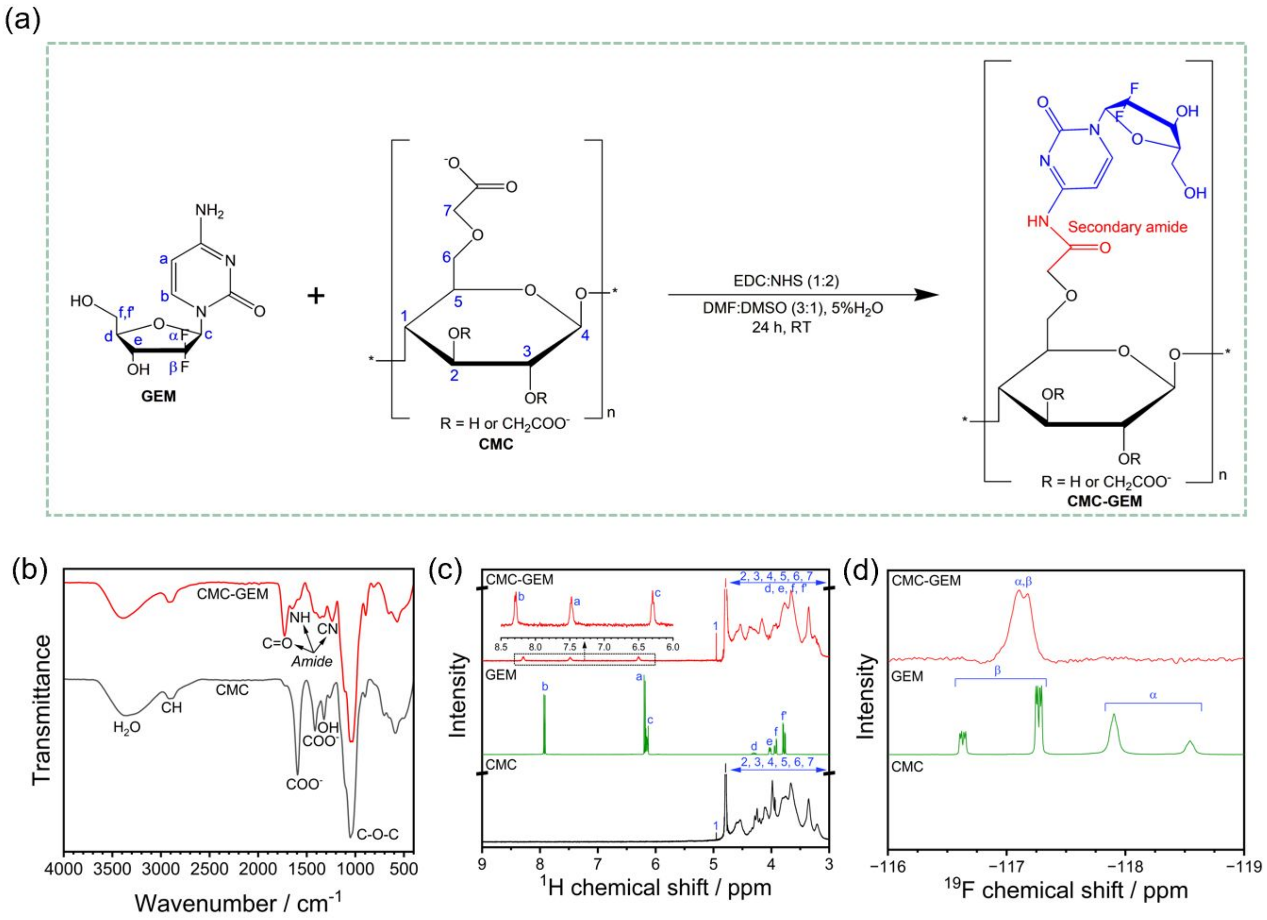

Figure S1. (a) Conjugation reaction of GEM with CMC via EDC-NHS coupling, (b) FTIR spectra, (c) ^{1}H NMR spectra (D_2O), (d) ^{19}F NMR spectra (D_2O).

S1. CMC-GEM Quantifications

The amount of GEM conjugated per milligram of CMC, denoted as *w*(GEM) per mg of NPs (μg mg^{-1}), was estimated based on the concentrations of GEM and CMC according to the following equation:

$$w(\text{GEM}) \text{ per mg of CMC} = \frac{w(\text{GEM})}{w(\text{CMC})} \qquad \text{(Equation S1)}$$

where *w*(GEM) (μg mL^{-1}) was determined by UV-Vis analysis after complete hydrolysis of the amide bond with the polymer, using the calibration curve for free GEM at pH 3.5 (Fig. S2(a)), and *w*(CMC) was 2 mg mL^{-1}.

The CMC/GEM molar ratio was calculated by converting w(CMC) of 2 mg mL^{-1} to molarity using the molar mass of 90000 g mol^{-1}, and w(GEM) converted to molarity using the molar mass of 263.198 g mol^{-1}.

The degree of substitution (DS) of CMC carboxyl groups by GEM molecules was estimated using the following equation:

$$DS = \frac{w(\text{GEM}) \times M(\text{CMC monomer})}{w(\text{CMC}) \times M(\text{GEM}) \times 0.7} \qquad \text{(Equation S2)}$$

where w(GEM) and w(CMC) are expressed in g mL^{-1}, M(GEM) is the molar mass of GEM, and M(CMC monomer) is the molar mass of the CMC monomer unit, equal to 202.77 g mol^{-1}.

2. Concentration of nanoparticles

The concentration of nanoparticles in µg mL^{-1}, denoted as w(NPs), was estimated based on the calcium content corresponding to the CaP core. To this end, the calcium concentration was first determined by atomic absorption spectroscopy (AAS). Prior to analysis, the NPs were digested in in 1 M HCl to ensure complete dissolution and release of calcium ions (Ca^{2+}) into solution. The resulting calcium concentration, denoted as $w(Ca^{2+})$ (µg mL^{-1}), was used to calculate the total amount of CaP core, assuming a stoichiometric composition of hydroxyapatite (HA, $Ca_{10}(PO_4)_6(OH)_2$) with a molar mass M(HA) of 1004.623 g mol^{-1}. Considering the molar mass of elemental calcium, M(Ca) = 40.078 g mol^{-1}, the w(NPs) was calculated using the following equation:

$$w(\text{NPs}) = w(\text{Ca}^{2+}) \times \frac{M(\text{HA})}{M(\text{Ca})} \qquad \text{(Equation S3)}$$

3. Number of nanoparticles

The number of nanoparticles per milliliter (NP mL^{-1}), denoted as N(NPs), was determined based on the previously calculated w(NPs) value and the volume of a single CaP core. Assuming a spherical morphology for the CaP core, with the radius (r) taken as half of the mean core diameter

determined by SEM analysis, and using a HA density denoted as ρ(HA) = 3.14 × 10^{-15} µg nm^{-3}, N(NPs) was calculated according to the following expression:

$$N(\mathrm{NPs}) = \frac{w(\mathrm{NPs})}{\frac{4}{3}\pi r^3 \cdot \rho(\mathrm{HA})} \qquad \text{(Equation S4)}$$

4. Quantifications of GEM loaded on nanoparticles

Based on the w(GEM) loaded on the NPs (µg mL^{-1}), determined by UV–Vis analysis of aliquots prepared by dissolving the pellets of NPs in 0.125 M HCl and applying the corresponding calibration curve in this medium (Fig. S2(b)), three parameters were estimated: the number of GEM molecules per NP, the amount of GEM per milligram of NPs, and the loading efficiency.

The number of GEM molecules per nanoparticle (molecule NP^{-1}), denoted as N(GEM), was calculated based on the previously determined value of N(NPs) and the number of GEM molecules loaded per milliliter, obtained by converting the w(GEM) to mol mL^{-1} using M(GEM), and multiplying by Avogadro's constant (N_A = 6.022 × 10^{23} mol^{-1}). Accordingly, N(GEM) per NP was calculated as follows:

$$N(\mathrm{GEM}) = \frac{\left(\frac{w(\mathrm{GEM}) \times 10^{-6}}{M(\mathrm{GEM})}\right) \cdot N_A}{N(\mathrm{NPs})} \qquad \text{(Equation S5)}$$

The amount of GEM loaded per milligram of NPs, denoted as GEM per mg of NPs (µg mg^{-1}), was estimated using the concentrations of GEM and NPs, w(GEM) and w(NPs), respectively, both expressed in µg mL^{-1}, according to the following relationship:

$$w(\mathrm{GEM})\ \text{per mg of NPs} = \frac{w(\mathrm{GEM})}{w(\mathrm{NPs})} \times 1000 \qquad \text{(Equation S6)}$$

The GEM loading efficiency (%) was determined based on the concentration of conjugated-GEM present after mixing the solutions containing the calcium precursor, the phosphate precursor, and the CMC-GEM conjugate. This calculation considered the final concentration of conjugated-GEM

under these conditions, which was approximately 10 μg mL^{-1}, and *w*(GEM) loaded on the NPs, according to the following equation:

$$\text{GEM loading efficiency (\%)} = \frac{\text{initial GEM concentration}}{w(\text{GEM})} \times 100 \qquad \text{(Equation S7)}$$

5. Determination of CMC loaded on nanoparticles

The concentration of CMC loaded on the NPs, denoted as *w*(CMC) in μg mL^{-1}, was estimated based on the amount of GEM loaded, *w*(GEM), and the quantity of GEM (μg) conjugated per mg of CMC, according to the following relation:

$$w(\text{CMC}) = \frac{w(\text{GEM})}{\text{GEM per mg of CMC}} \times 1000 \qquad \text{(Equation S8)}$$

The number of CMC chains per NP and the amount of CMC per milligram of NPs, denoted as *N*(CMC) and *w*(CMC) per mg of NPs, were calculated similarly to the procedure employed for GEM (equations 6 and 7), but using the molar mass of CMC (90000 g mol^{-1}).

6. Quantifications of FA conjugated on nanoparticles

The number of FA molecules per NP, denoted as *N*(FA), and the amount of FA per milligram of NPs, denoted as *w*(FA) per mg of NPs, were estimated using a methodology similar to that applied for GEM (equations 6 and 7). However, in this case, the calculation was based on the amount of FA loaded onto the NPs (*w*(FA) in μg mL^{-1}), determined by UV–Vis analysis of aliquots prepared by dissolving the pellets of NPs in 0.166 M HCl and applying the corresponding calibration curve in this medium (Fig. S3(a,b)), and the molar mass of folic acid (441.40 g mol^{-1}).

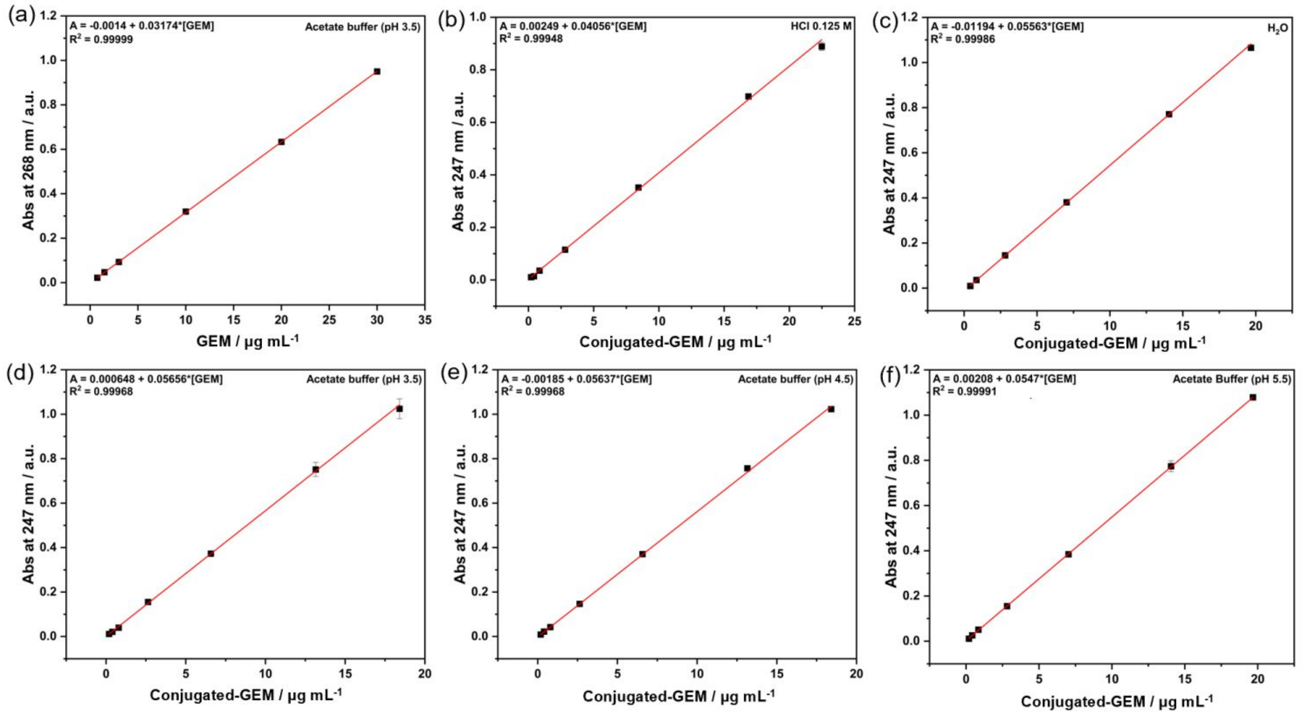

Figure S2. Calibration curves obtained by UV–Vis spectroscopy: (a) free GEM in acetate buffer (pH 3.5); (b) CMC-GEM in 0.125 M HCl; (c) CMC-GEM in water; (d) CMC-GEM in acetate buffer (pH 3.5); (e) CMC-GEM in acetate buffer (pH 4.5); and (f) CMC-GEM in acetate buffer (pH 5.5).

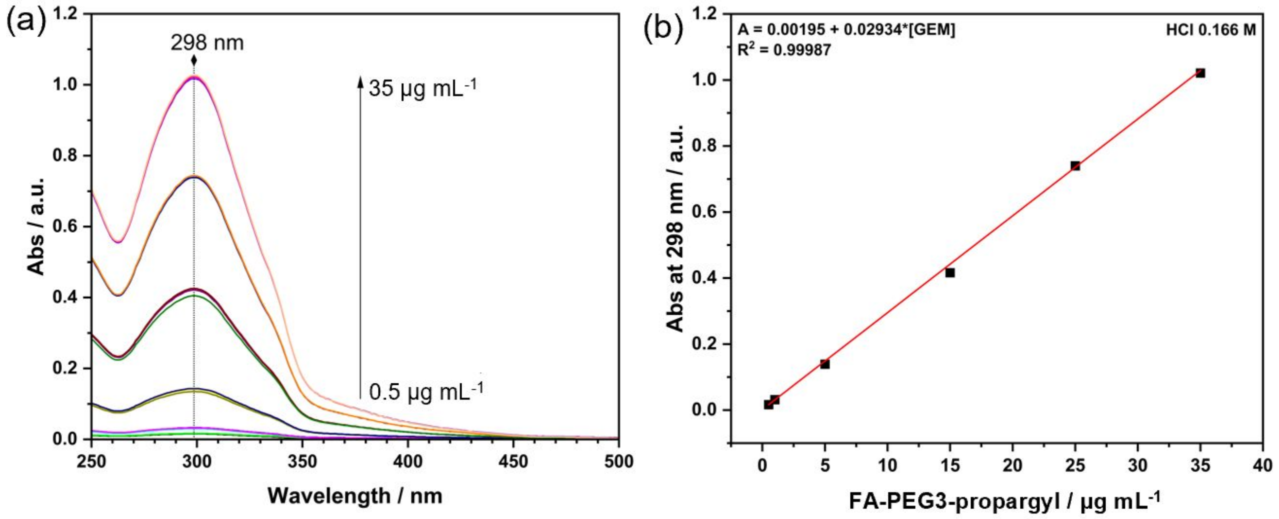

Figure S3. (a) UV–Vis absorption spectra of Folate-PEG3-Propargyl at different concentrations in 0.166 M HCl, and (b) corresponding calibration curve.

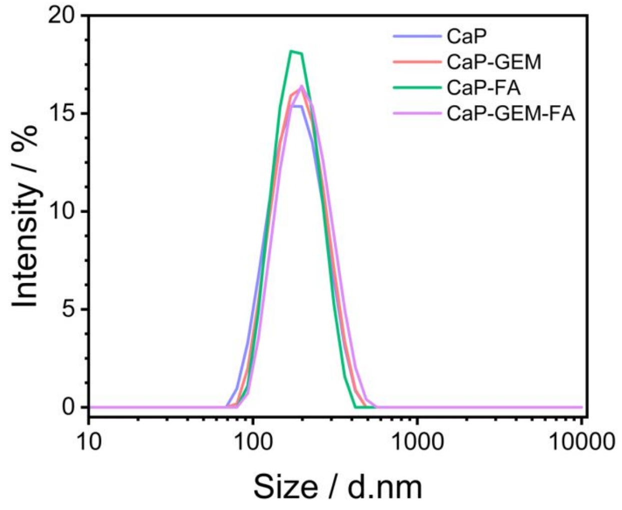

Figure S4. Correlation between particle size and signal intensity obtained by DLS.

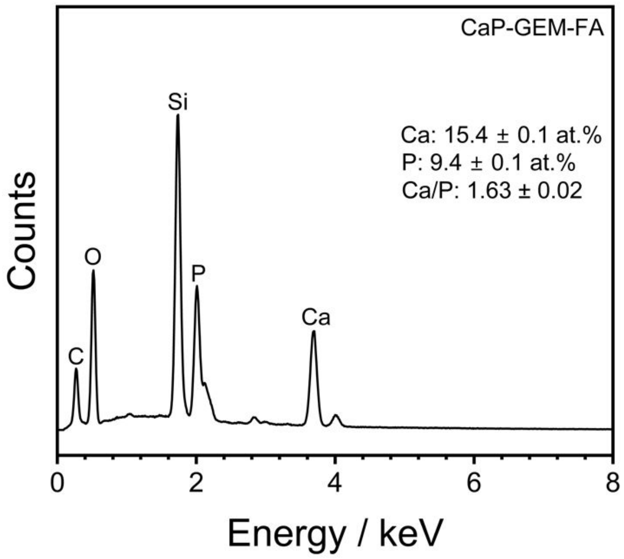

Figure S5. Elemental analysis of CaP-GEM-FA NPs by EDS.

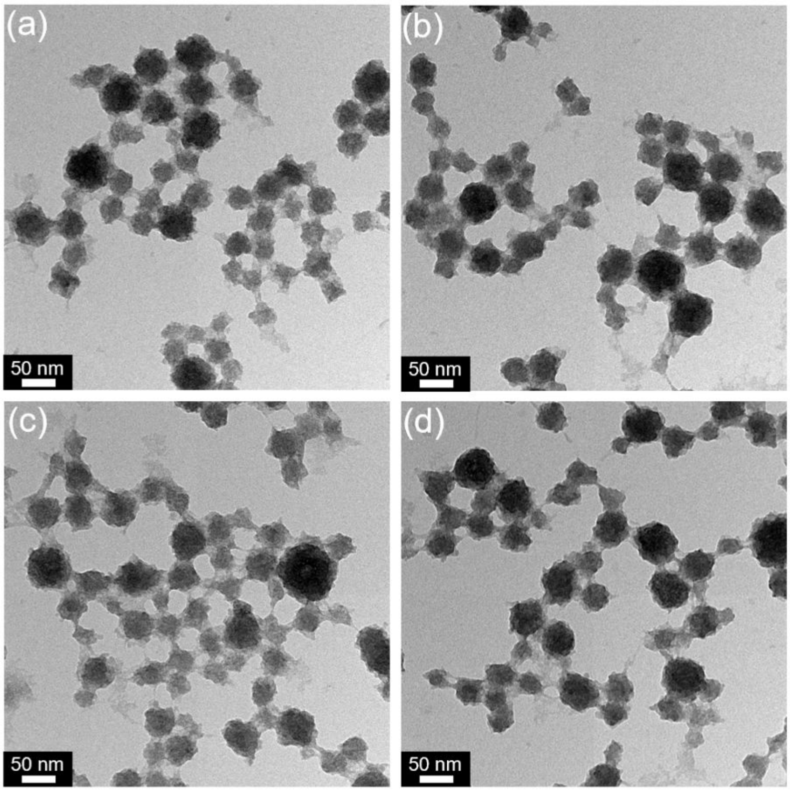

Figure S6. TEM images of (a) CaP, (b) CaP-GEM, (c) CaP-FA, and (d) CaP-GEM-FA NPs.

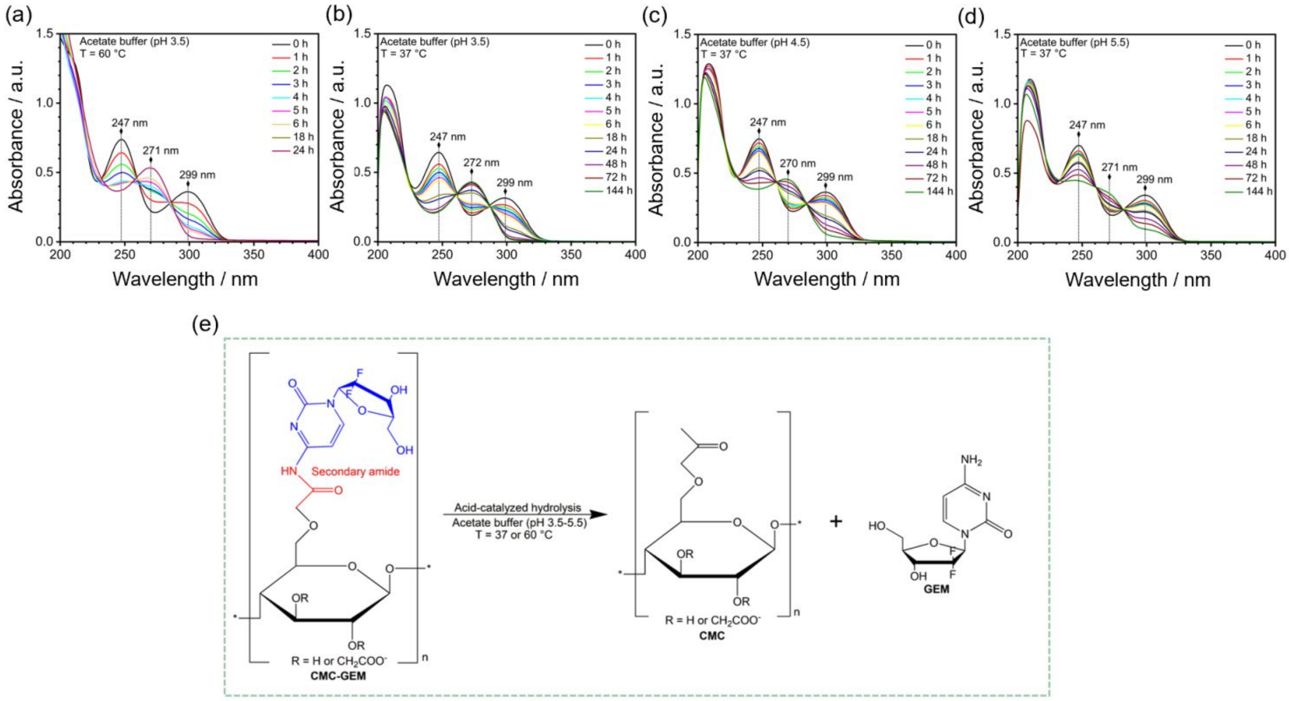

Figure S7. Kinetic study of the acid-catalyzed hydrolysis of the secondary amide group in CMC-GEM conjugated polymer. (a-d) UV-Vis spectra recorded at different time intervals after exposure to acetate buffer (pH 3.5, 4.5, and 5.5) at 60 °C or 37 °C, used to quantify the amount of conjugated GEM and to demonstrate the pH-responsive behavior of CMC-GEM, simulating the acidic intracellular environment of endolysosomal vesicles. (e) Proposed hydrolysis reaction for CMC-GEM.

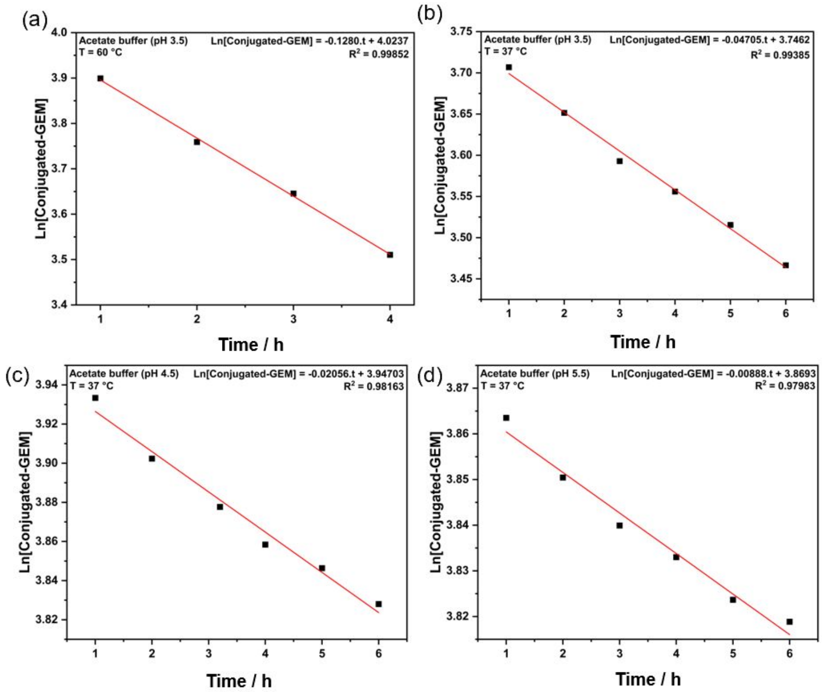

Figure S8. Linearities used to estimate the rate constants and half-life of CMC hydrolysis in acetate buffer: (a) pH 3.5 at 60 °C, (b) pH 3.5 at 37 °C, (c) pH 4.5 at 37 °C, and (d) pH 5.5 at 37 °C.

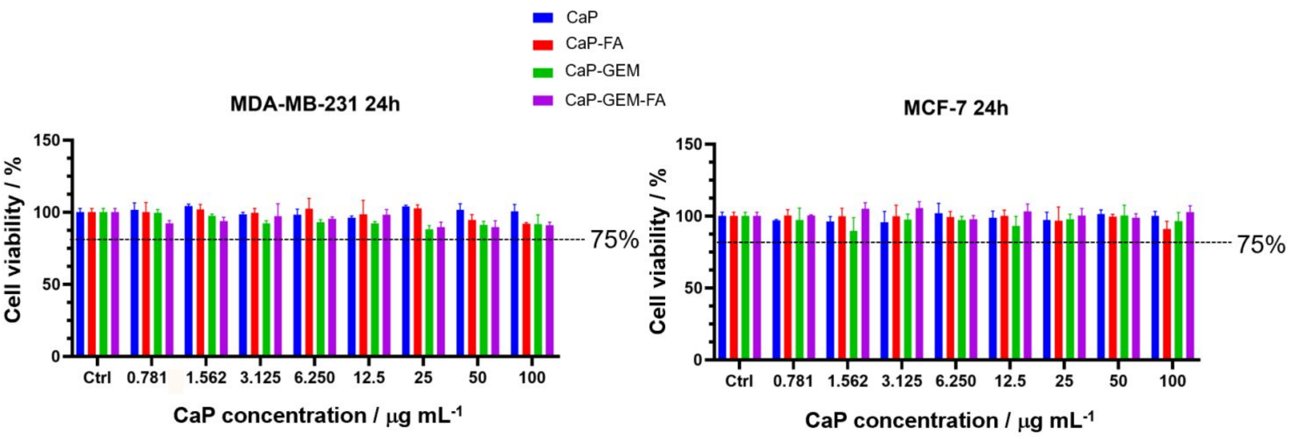

Figure S9. MTT assay of CaP NPs after 24 h of incubation in MDA-MB-231 and MCF-7 cells.

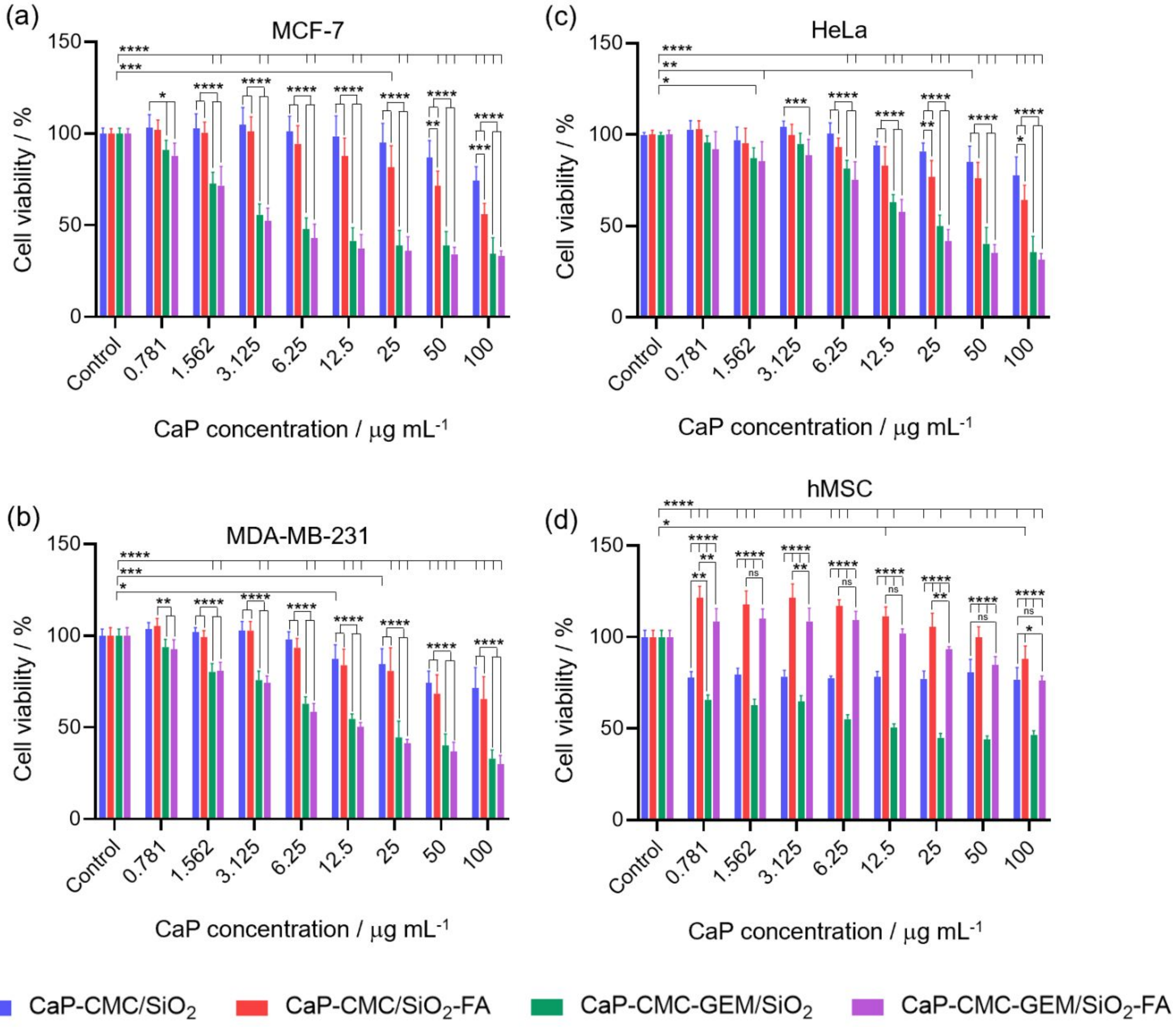

Figure S10. (a–d) MTT assay results for all cell lines in the present study. Significance levels were set at $p < 0.05$ (*), $p < 0.01$ (**), $p < 0.001$ (***), and $p < 0.0001$ (****).
